# Joint Exploration of Favorable Haplotypes for Mineral Concentrations in Milled Grains of Rice (*Oryza sativa* L.)

**DOI:** 10.3389/fpls.2018.00447

**Published:** 2018-04-12

**Authors:** Guo-Min Zhang, Tian-Qing Zheng, Zhuo Chen, Yong-Li Wang, Ying Wang, Yu-Min Shi, Chun-Chao Wang, Li-Yan Zhang, Jun-Tao Ma, Ling-Wei Deng, Wan Li, Tian-Tian Xu, Cheng-Zhi Liang, Jian-Long Xu, Zhi-Kang Li

**Affiliations:** ^1^Northern Japonica Rice Molecular Breeding Joint Research Center, Chinese Academy of Sciences, Haerbin, China; ^2^Institute of Crop Sciences, National Key Facility for Crop Gene Resources and Genetic Improvement, Chinese Academy of Agricultural Sciences, Beijing, China; ^3^Institute of Genetics and Developmental Biology, Chinese Academy of Sciences, Beijing, China; ^4^Guangxi Key Laboratory of Rice Genetics and Breeding, Rice Research Institute, Guangxi Academy of Agricultural Sciences, Nanning, China; ^5^Shenzhen Institute of Breeding for Innovation, Chinese Academy of Agricultural Sciences, Shenzhen, China

**Keywords:** favorable haplotype joint exploration, grain mineral concentration, GMC, quantitative trait locus, QTL, milled grain, biofortification molecular breeding, rice (*Oryza sativa* L.)

## Abstract

Grain minerals in rice, especially those in milled grains, are important sources of micro-nutrition elements, such as iron (Fe), zinc (Zn), manganese (Mn), copper (Cu), and selenium (Se), and of toxic heavy metal elements, especially cadmium (Cd), for populations consuming a rice diet. To date, the genetic mechanism underlying grain mineral concentrations (GMCs) in milled grain remains largely unknown. In this report, we adopted a set of 698 germplasms consisting of two subsets [*indica*/*Xian* (X-set) and *japonica*/*Geng* (G-set)], to detect quantitative trait loci (QTL) affecting GMC traits of Fe, Zn, Cd, Mn, Cu, and Se in milled grains. A total of 47 QTL regions, including 18 loci and 29 clusters (covering 62 Cd loci), responsible for the GMCs in milled grains were detected throughout the genome. A joint exploration of favorable haplotypes of candidate genes was carried out as follows: (1) By comparative mapping, 10 chromosome regions were found to be consistent with our previously detected QTL from linkage mapping. (2) Within eight of these regions on chromosomes 1, 4, 6, 7, and 8, candidate genes were identified in the genome annotation database. (3) A total of 192 candidate genes were then submitted to further haplotype analysis using million-scale single nucleotide polymorphisms (SNPs) from the X-set and the G-set. (4) Finally, 37 genes (19.3%) were found to be significant in the association between the QTL targeting traits and the haplotype variations by pair-wise comparison. (5) The phenotypic values for the haplotypes of each candidate were plotted. Three zinc finger (like) genes within two candidate QTL regions (*qFe6-2* and *qZn7*), and three major GMC traits (Fe, Zn, and Cd) were picked as sample cases, in addition to non-exhausted cross validations, to elucidate this kind of association by trait value plotting. Taken together, our results, especially the 37 genes with favorable haplotype variations, will be useful for rice biofortification molecular breeding.

## Introduction

Micronutrient malnutrition (or “hidden hunger”) is widespread throughout different countries (Kumssa et al., 2015), especially among poor populations whose daily caloric intake is confined to staple cereals (Gregorio and Htut, 2003; Ma et al., 2008; Bhullar and Gruissem, 2013). The development of biofortified cereals, especially mineral-dense rice, remains an efficient way to alleviate malnutrition in developing countries worldwide, including China (Gregorio and Htut, 2003; De Steur et al., 2012). Meanwhile, as a side effect of modernization, heavy metal pollution of arable land has become more and more severe; concentrations of toxic minerals, especially cadmium (Cd), are increasing in cereal grains, which threatens human health (Al-Saleh and Shinwari, 2001; Huang et al., 2007; Fu et al., 2008; Hang et al., 2009). Currently, with the fast expansion of rice cultivation to Northeast China, the grain mineral concentrations (GMCs) in early-matured *japonica*/*Geng* type rice have become more and more important in rice production.

In addition to its relatively small genome, rice remains the world's most popular staple food crop (Dawe et al., 2010; GRiSP, 2013); therefore, both the biofortification and the relief of heavy metal pollution in rice have attracted increased research attention. The GMCs belong to complex traits controlled by multiple quantitative trait loci (QTL). Some QTL mapping studies have been carried out with different populations (Tang, 2007; Lu et al., 2008; Shen et al., 2008; Garcia-Oliveira et al., 2009; Zhang et al., 2009, 2011, 2014; Zhong, 2010; Anuradha et al., 2012; Bekele et al., 2013; Du et al., 2013; Kumar et al., 2014; Norton et al., 2014; Huang et al., 2015; Nawaz et al., 2015; Hu et al., 2016), and *in-silico* mapping (Chandel et al., 2011) for the GMCs in brown rice has been performed. GMC-related QTL tend gather in four regions on chromosomes 2, 3, 4, 6, 7, and 11, respectively. Specifically, there are three regions gathering QTL controlling Cd concentration in rice grains on chromosomes 4, 7, and 11, among which, the one on chromosome 7 is supported by evidence from four different tests. The single causative gene was identified as *OsNramp1* (Ueno et al., 2009a,b, 2010; Ishikawa et al., 2010; Tezuka et al., 2010; Abe et al., 2011). However, just as the other cloned genes identified as associated with GMCs, such as, *OsVIT* (Zhang et al., 2012) and *OsNAS* (Lee et al., 2009) for Fe, *OsLCT1* (Uraguchi et al., 2014) and *OsHMA3* (Ueno et al., 2010) for Cd, *OsNramp5* (Ishimaru et al., 2012; Liu et al., 2017; Tang et al., 2017) for Mn, and *OsHMA4* (Huang et al., 2016) for Cu, it's also mainly responsible for the GMCs in the aleuronic layer rather than the endosperm, which is the major part of the milled grain. Currently, attempts have been made by a few molecular biologists using endosperm-specific promoters to improve the GMCs in milled grains (Zheng et al., 2010; Masuda et al., 2012). However, the genetic mechanism of GMCs in milled grains remains largely unknown.

Previously, we used two sets of backcrossed inbred lines (BILs) derived from the same donor, and two elite new varieties in Southwestern China, Ce258 and Zhongguangxiang1 (ZGX1) as recipients, to assess the genetic background and the genotypic by environment (G × E) effects of GMC traits in rice milled grains using QTL mapping (Xu et al., 2015). Therefore, in the present study, QTL information from that linkage mapping work was used to confirm the results of a genome-wide association study (GWAS) using a set of 698 sequenced germplasms. Favorable haplotype joint exploration for candidate genes within important QTL regions was also carried out.

## Materials and methods

### Plant materials and field experiments

A set of 698 germplasms was adopted in this study. The set comprised two subsets, one was an *indica*/*Xian* subset (X-set) including 265 accessions randomly chosen from the 3K genome project (The 3,000 Rice genomes project, 2014), and the other was an early *japonica/Geng* subset (G-set), which included 433 accessions with sequencing data from similar sequencing pipelines. According to their maturation times, the X-set was planted at Sanya (18.3°N, 109.3°E) of Hainan province and the G-set was planted at Haerbin (45.8°N, 126.65°E) of Heilongjiang province. A small set of accessions was used as a control panel to check the variances between different environments.

All of the above plant materials were transplanted into the field at a spacing of 13.2 cm between individuals and 25 cm between rows, with a final planting density of approximately 18,000 individuals per 667 m^2^. Field management was carried out according to the local farmers' practice. At the mature stage (about 40 days after flowering), seeds were bulk-harvested for each line. The seeds were air-dried and stored for 3 months in a drying house before being evaluated for the mineral concentrations (GMCs) in the milled grains.

Basic physical and chemical properties of the soil in the paddy field were analyzed using routine analytical methods of agricultural chemistry (Lu, 1999).

### Evaluation of grain mineral concentrations (GMCs)

Dried seeds of each line were de-hulled, polished and then milled into flour, according to the surging and grind-milling method described in our previous report (Xu et al., 2015), to prevent possible mineral contamination, especially by Fe. About 0.3 g of rice flour was digested with 6 ml of HNO_3_ and 0.2 ml of H_2_O_2_ using a microwave digestion system (Microwave300, Anton PAAR, Graz, Austria), with the following parameters: 5 min at 700 W, 700–1,200 W for 10 min, and 1,200 W for 20 min. The samples were then transferred to a block heater at 160°C for further digestion. The remaining 1 ml of digested sample was diluted with 50 ml of Milli-Q water before analysis. The Fe, Zn, Cd, Mn, Cu, and Se concentrations in the digested samples were determined using the methods described in our previous report (Xu et al., 2015). Two standards and two controls were set in each testing batch. Three replications of the tests were performed for each sample.

### Genotyping by sequencing and shared SNP extraction

The X-set germplasms were re-sequenced with an averaged depth of more than 10× (The 3,000 Rice genomes project, 2014). The cleaned reads were then mapped to the reference genome of Nipponbare (IRGSP1.0), and about 14 M high-quality single nucleotide polymorphisms (SNPs) were identified (The 3,000 Rice genomes project, 2014). Based on these 14 M SNP, a set of 2.9 M SNPs related to potential protein-coding areas was carefully selected. To build an SNP set for primary association studies, a subset of about 27,921 SNPs was selected from the 2.9 M SNPs by choosing one SNP per 100 counts, as described in our previous GWAS mapping work (Zhang et al., 2016). For the G-set germplasms, the quantity of the full set of SNPs was about 4 M. Finally, about 13 K SNP markers shared by both sets were extracted and submitted for further analyses, including sample clustering, principal component analysis (PCA), and GWAS mapping. These analyses were also carried out with the X-set and G-set data independently and compared with the pooled data. To perform deeper mining, favorable haplotypes were jointly explored for candidate genes within important QTL regions, based with the original 14 M and 4 M SNPs in the X-set and G-set, respectively.

### Data analysis, QTL mapping, and haplotype analysis

Basic statistical analysis of the GMC traits, including the analysis of variance (ANOVA) and Duncan's *t*-test, were conducted using SAS software (S. A. S. I. Inc., 2004). The basic scenario of a compressed mixed linear model (Zhang et al., 2010), implemented in the Genomic Association and Prediction Integrated Tool (GAPIT) Version 2 (Lipka et al., 2012), was adopted for association analysis between QTL-flanking markers and GMC traits for the pooled, the X-set, and the G-set. Parameters for GAPIT were set with reference to our previous report (Zhang et al., 2016). A relatively stringent threshold was adopted to identify significant correlations between the SNPs and GMC traits, comprising a −log_10_(*P*)-value of 6.0. To minimize to the possibility of type II errors in QTL detection (Li, 2001), a relatively loose threshold of 3.0 was adopted for the loci with supporting evidence from our previous linkage mapping report (Xu et al., 2015) or other references. The allelic effects were estimated by setting the Major.allele.zero = TRUE in GAPIT Version 2 to identify the donors of favorable alleles and their effects on GMC traits.

Subsequently, a joint exploration of favorable haplotypes was carried out according to the following steps: (1) By comparative mapping, we compared the results from the association mapping with the linkage mapping results from our previous report (Xu et al., 2015). The regions containing the jointly detected QTLs were then subjected to candidate gene analysis. (2) We searched the regions in the annotation dataset with wet-lab supporting evidence from the Rice Annotation Project database (RAP-DB) (Ohyanagi et al., 2006). (3) We then screened the genes by annotation information. If there were any obvious supporting evidence from the functional annotation, representing the relationships between the gene and the QTL targeting trait, then these genes would be highly focused in the next step. (4) Next, we compared all the mean values of the targeting traits for all the haplotypes of each candidate gene using pair-wise comparisons with Duncan's *t*-test to identify significant associations between the variations of haplotypes and the QTL targeting traits. (5) Finally, we plotted the QTL targeting trait values for the haplotypes of each candidate in a straight-forward view. This joint haplotype exploration of the candidate genes was performed with the aid of Perl scripts and the full sets of SNPs in the X-set and G-set, respectively. For graphing and plotting, both Excel and R scripts were used.

## Results

### Performance of the 698 sequenced accessions

**Figure 1:**
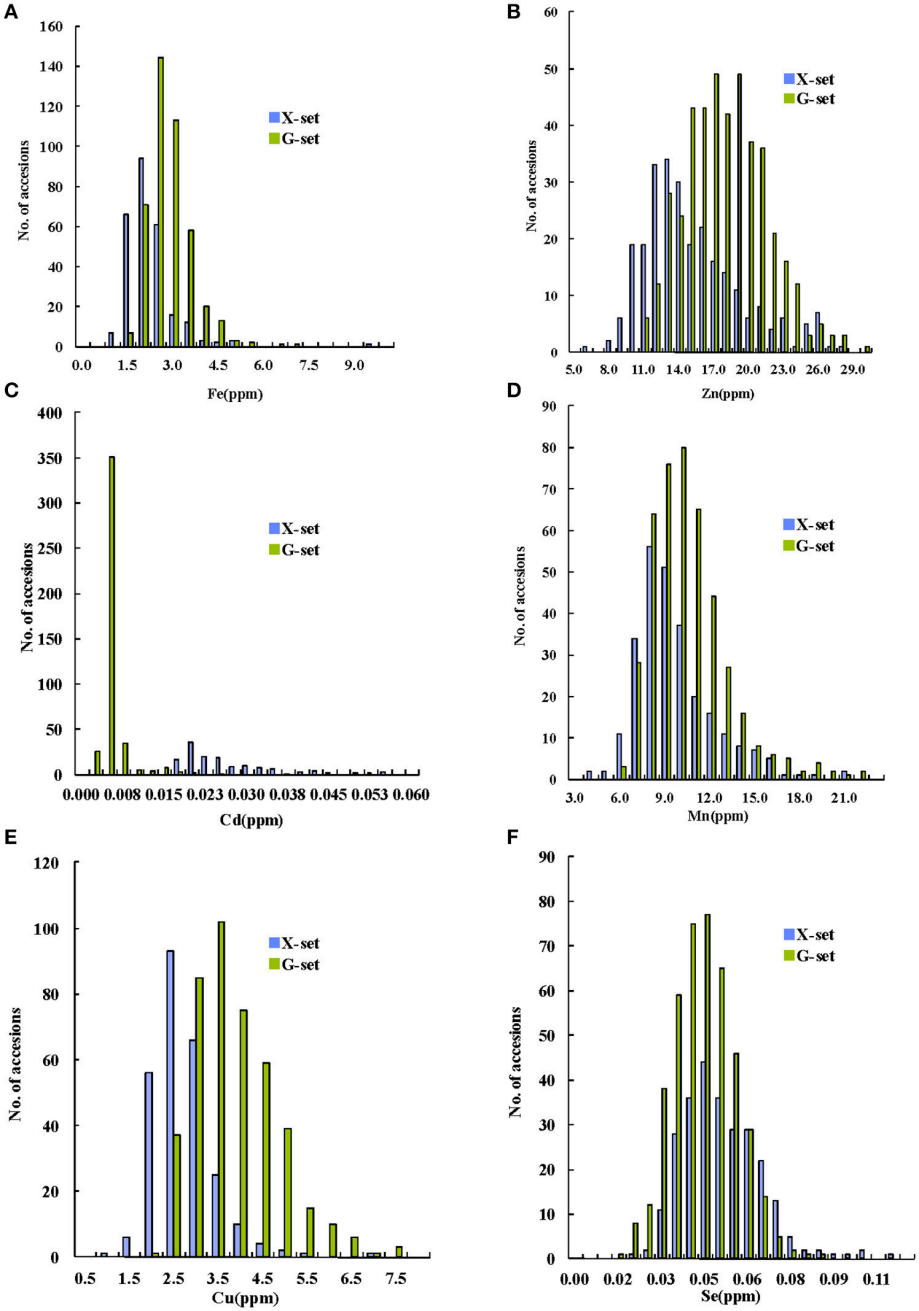
Distribution of grain mineral concentrations (GMCs) in a set of 698 sequenced germplasms with two subsets (X(ian/indica)-set and G(eng/japonica)-set). **(A–F)** Distribution graphs for Fe, Zn, Cd, Mn, Cu, and Se concentrations in milled grains, respectively.

Among the 698 sequenced accessions, a wide range of variation was found for the GMC traits in the milled grains. As shown in Figures 1A–C, the concentrations of three major GMC traits (Fe, Zn, and Cd) ranged from 0.9 to 9.1 ppm, 5.8 to 29.6 ppm, and 0.002 to 0.054 ppm, with mean values of 2.4, 16.4, and 0.009 ppm, respectively. The concentrations of the other three GMC traits (Mn, Cu, and Se) (Figures 1D–F) ranged from 3.6 to 22.0 ppm, with a mean value of 9.7 ppm; from 0.8 to 7.5 ppm, with a mean value of 3.2 ppm; and from 0.01 to 0.11 ppm, with a mean value of 0.04, ppm, respectively. All the GMC traits fitted normal or normal-like distributions in the pooled set, except for the Cd concentration, which showed a binomial-like distribution (Figure 1). Notably, when we highlighted samples from the X-set and G-set with different colors, a major proportion of G-set samples were found to have higher Zn and Cu, but lower Cd concentrations. For the other three GMC traits, the phenotypic value distributions between the two sets overlapped markedly, especially for the Se concentration. The affects on the GMC trait values were caused by multiple factors, including different environmental conditions, especially the soil (Supplementary Table 1), as well as the genetic factors, were much more complex than we expected. Nevertheless, according to the ANOVA results based on the control panel (Supplementary Table 2), all the genotypic variances showed higher statistical significances than the environmental variances. Although limited by the diversity of the control panel, the effects of the genotypic variances for most GMC traits were only marginally significant or insignificant, except for the Zn and Mn concentrations.

**Figure 2:**
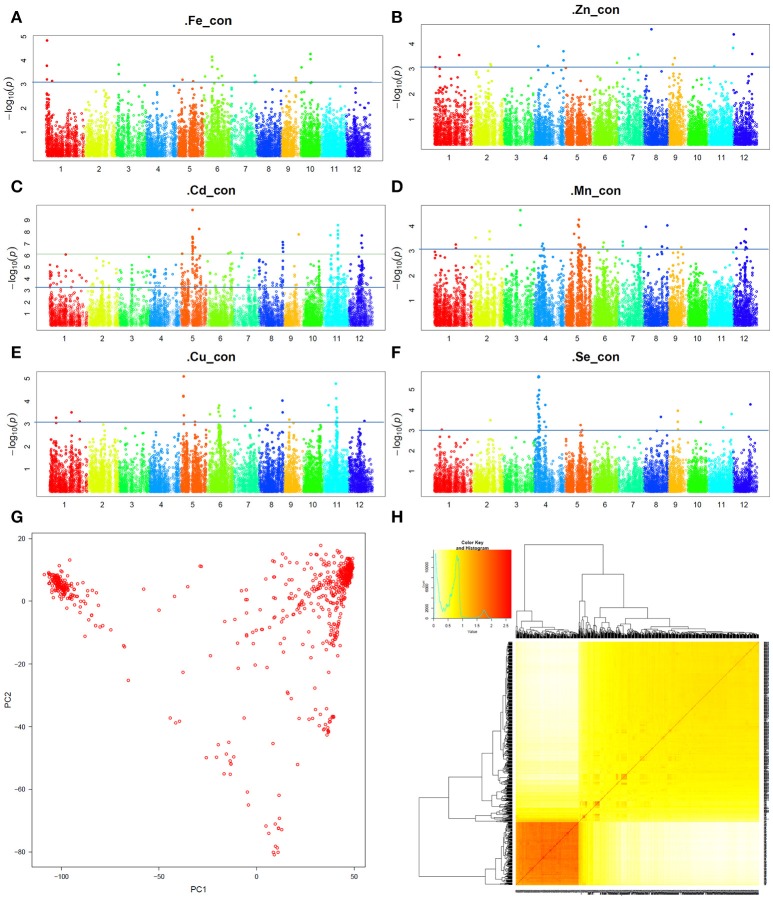
Genome-wide association study (GWAS) results for grain mineral concentrations (GMCs) in a set of 698 sequenced germplasms. **(A–F)** Manhattan plot for the GWAS results for Fe, Zn, Cd, Mn, Cu, and Se concentrations in milled grains, respectively; **(G)** Principal component analysis (PCA) plots based on the single nucleotide polymorphism (SNP) genotyping data; **(H)** VanRaden map for the Kinship of the 698 germplasms.

The Manhattan plots presenting the GWAS mapping results of the six GMC traits were shown in Figures 2A–F. Sample clustering and PCA analyses were also carried out based on the 13 K SNPs. The PCA result for the pooled data is shown in Figure 2G, and the kinship between the 698 accessions is presented in Figure 2H. For comparison, the PCA results obtained from the X-set and G-set independently are also shown in the Supplementary Figure 1. The results showed that the segregating pattern of the pooled set was quite similar to that of the X-set, whereas the G-set seemed relatively uniform. Considering that the optimum setting of the PCA value might vary according to different GMC traits, during the GWAS analysis with GAPIT, the Model.selection was set as TRUE for the optimum PCA value setting.

### Identification of loci controlling the six GMC traits

According to the comparisons between the GWAS results from the pooled set and the two independent sets (X-set and G-set) shown in Supplementary Figures 2–7, a compensating mode was found between them. This meant that most signals in the pooled set were donated by either the X-set or the G-set, although the significance levels of the signals in the pooled set would be somewhat reduced if they were not significant in both subsets. To focus on the GMC QTL throughout different populations (also termed genetic background independent) and environments (also termed stably expressed), we adopted the results from the analyses based on the pooled set for further joint exploration of favorable haplotypes. A total of 47 QTL regions, including 18 loci and 29 clusters covering 62 Cd loci (Table 1, Figure 2) were detected by GWAS mapping for the six GMC traits from these 698 sequenced accessions. They included six loci for Fe, four loci for Zn, three loci for Mn, two loci for Cu, three loci for Se, and 62 loci belonging to 29 clusters for the Cd concentration. The average –log_10_ value for these loci was 5.2 (range, 3.1–9.9). The –log_10_ values varied by different GMC traits: It was 4.0 for Fe (range, 3.1–4.8), 3.5 for Zn (range, 3.4–3.6), 5.5 for Cd (range, 3.2–9.9), 4.3 for Mn (range, 4.0–4.6), 5.0 for Cu (range, 4.8–5.1), and 4.5 for Se (range, 3.8–5.6).

**Table 1:** Quantitative trait loci (QTL) affecting grain mineral concentrations (GMCs) detected by a genome-wide association study (GWAS) in a panel of 698 germplasms.

**Trait**	**Clst^a^**	**Loci**	**Ch**	**Range**	**−log_10_**	**FAE^b^**	**QTL reported^c^**	**Ref^d^**
Fe		*qFe1*	1	841,961~959,278	4.8	0.300		
		*qFe3*	3	4,145,494~4,182,173	3.8	−0.263		
		*qFe6-1*	6	10,055,520~10,274,263	4.2	−0.158		
		*qFe6-2*	6	16,404,065~21,506,253	3.6	0.320	*qFe6*	
		*qFe7*	7	27,770,508~27,788,464	3.1	0.148	*qFe7*	
		*qFe10*	10	10,945,859~11,075,230	4.3	0.337		
Zn		*qZn1*	1	6,179,574~6,204,400	3.5	−0.890		id1005056-58 (Norton et al., 2014)
		*qZn7*	7	22,891,126~26,101,517	3.6	−0.680	*qZn7*	*qZn7* (Huang et al., 2015; Hu et al., 2016);*qZN-7* (Lu et al., 2008); id7003641 (Norton et al., 2014)
		*qZn9*	9	5,174,170~7,387,104	3.4	1.795		
		*qZn12*	12	21,887,797~21,913,241	3.6	−0.398	*qZn12*	
Cd	Clst1a	*qCd1-1*	1	212,589~434,398	5.2	−0.002	*qCd1*	
		*qCd1-2*	1	1,603,456~1,905,348	4.5	0.003	*qCd1*	
	Clst1b	*qCd1-3*	1	8,542,202~18,485,590	6.1	−0.002		
	Clst2a	*qCd2-1*	2	10,199,643~18,518,546	5.8	−0.002		
	Clst2b	*qCd2-2*	2	25,207,241~33,640,277	5.4	−0.002		*qCd2b* (Zhang et al., 2014)
	Clst3a	*qCd3-1*	3	1,652,156~2,158,456	4.7	0.002		*qCd3* (Zhang et al., 2014)
		*qCd3-2*	3	3,337,100~3,355,424	4.5	−0.002		
	Clst3b	*qCd3-3*	3	15,185,771~29,212,237	5.2	0.003		
		*qCd3-4*	3	29,214,304~32,538,230	4.7	0.003		
		*qCd3-5*	3	32,638,170~35,155,759	5.9	−0.002		*qCd3* (Huang et al., 2015)
	Clst4a	*qCd4-1*	4	1,112,387~2,047,665	4.4	−0.001		
		*qCd4-2*	4	4,734,877~5,034,318	4.2	0.003		
		*qCd4-3*	4	5,302,854~5,801,556	4.8	−0.002		
		*qCd4-4*	4	6,224,157~6,246,104	4.6	0.002		
	Clst4b	*qCd4-5*	4	16,868,613~16,869,822	4.4	−0.001		
		*qCd4-6*	4	18,424,682~18,529,758	4.8	0.002		
	Clst4c	*qCd4-7*	4	26,418,529~30,460,722	5.2	0.003	*qCd4*	*qCd4-2* (Kashiwagi et al., 2009)
	Clst5a	*qCd5-1*	5	1,142,167~3,242,916	6.2	−0.002		*qCd5* (Zhang et al., 2014)
		*qCd5-2*	5	4,859,767~4,870,282	4.6	0.002		
	Clst5b	*qCd5-3*	5	8,264,080~8,297,556	4.7	−0.002		
	Clst5c	*qCd5-4*	5	13,797,802~14,052,508	9.9	−0.002		
		*qCd5-5*	5	14,065,017~14,071,607	4.5	0.002		
		*qCd5-6*	5	14,075,029~16,119,884	7.4	−0.002		
		*qCd5-7*	5	16,818,124~19,141,063	6.7	−0.002		*qCd5.1* (Huang et al., 2015)
	Clst5d	*qCd5-8*	5	21,486,695~23,456,509	8.3	−0.002		
	Clst6a	*qCd6-1*	6	4,365,001~4,400,366	4.5	0.004		
	Clst6b	*qCd6-2*	6	10,411,282~11,457,254	4.4	−0.002	*qCd6*	Segment_on_Chr6 (Ishikawa et al., 2005)
	Clst6c	*qCd6-3*	6	22,117,058~22,123,339	4.3	0.002		*OsLCT1* (Uraguchi et al., 2011)
	Clst6d	*qCd6-4*	6	27,586,307~27,591,921	4.9	−0.002		
		*qCd6-5*	6	27,919,935~27,938,490	6.3	0.003		
		*qCd6-6*	6	28,441,362~29,887,070	5.3	−0.002		*qCd6* (Zhang et al., 2014)
	Clst7a	*qCd7-1*	7	9,491,735~10,356,836	6.2	0.004		*qGCd7/qSCd7* (Ishikawa et al., 2010) *qCdp7* (Abe et al., 2011)
	Clst7b	*qCd7-2*	7	17,677,268~24,927,574	4.7	0.002		*qCDCN-7* (Shen et al., 2008)
	Clst8a	*qCd8-1*	8	098,858~736,546	5.6	−0.002	*qCd8*	Segment_on_Chr8 (Ishikawa et al., 2005)
	Clst8b	*qCd8-2*	8	4,494,409~7,760,106	5.4	−0.002		
	Clst8c	*qCd8-3*	8	24,758,957~26,561,629	4.3	−0.002		*qCd8* (Zhang et al., 2014)
		*qCd8-4*	8	27,252,563~27,275,319	7.1	0.003		
		*qCd8-5*	8	27,313,865~27,323,824	5.0	−0.002		
		*qCd8-6*	8	27,425,405~27,460,834	4.9	0.002		
		*qCd8-7*	8	27,501,982~27,582,900	6.9	−0.002		
	Clst9a	*qCd9-1*	9	12,135,431~12,165,192	5.1	−0.002		
		*qCd9-2*	9	12,405,421~17,243,659	7.8	0.004		
	Clst10	*qCd10-1*	10	8,425,690~14,527,032	5.3	−0.002		
		*qCd10-2*	10	16,043,069~18,482,561	5.5	−0.002		
	Clst11a	*qCd11-1*	11	6,233,769~6,354,200	3.2	0.001	*qCd11*	
		*qCd11-2*	11	8,096,875~8,953,846	7.7	−0.002		
		*qCd11-3*	11	9,162,686~9,204,553	5.3	0.002		
		*qCd11-4*	11	9,396,989~16,643,233	8.6	−0.002		
	Clst11b	*qCd11-5*	11	16,648,036~16,691,008	6.3	0.002		
		*qCd11-6*	11	16,774,292~16,850,863	6.9	−0.002		
		*qCd11-7*	11	16,915,560~20,181,453	5.1	0.002		*qCd11* (Kashiwagi et al., 2009)
		*qCd11-8*	11	23,757,657~23,977,853	4.4	−0.002		*qCd11* (Tang, 2007)
		*qCd11-9*	11	25,493,176~27,669,556	4.2	0.002		*qCd11* (Tang, 2007)
	Clst12a	*qCd12-1*	12	1,615,274~1,630,986	4.7	−0.002		
		*qCd12-2*	12	2,275,959~2,471,184	5.3	0.004		
		*qCd12-3*	12	2,956,398~8,288,204	4.0	−0.001		
	Clst12b	*qCd12-4*	12	11,270,271~11,777,847	5.9	0.004		
		*qCd12-5*	12	12,194,972~14,140,041	5.1	−0.001		
		*qCd12-6*	12	14,681,128~15,757,691	7.7	0.003		
		*qCd12-7*	12	15,804,848~15,872,325	5.2	−0.002		
		*qCd12-8*	12	16,987,855~17,467,228	4.9	0.002		
	Clst12c	*qCd12-9*	12	24,218,754~25,662,740	4.1	−0.001		*qSCd12* (Ishikawa et al., 2010)
Mn		*qMn3*	3	20,083,700~20,107,682	4.6	1.317		
		*qMn5*	5	15,026,427~15,945,778	4.3	−0.518		
		*qMn8*	8	27,194,754~27,212,235	4.0	0.943		
Cu		*qCu5*	5	3,343,234~3,704,085	5.1	−0.333		*qCu5* (Zhang et al., 2014)
		*qCu11*	11	14,314,371~14,705,331	4.8	0.262		
Se		*qSe4-1*	4	3,566,285~5,597,802	5.6	0.005		
		*qSe4-2*	4	11,568,849~12,645,615	4.2	0.004		
		*qSe11*	11	27,190,782~27,196,075	3.8	0.003		

a *QTL cluster*.

b *Favorable allele effect (FAE) values of the peak markers*.

c *A GMCs-related QTL detected by linkage mapping in our previous report (Xu et al., 2015), in which the three parents for the BC populations were also involved in our germplasms for the GWAS mapping*.

d *The number in brackets are reference codes as listed in reference section*.

Alleles from the germplasms increased the GMCs at about 38 (47.5%) of the above 80 loci, while they decreased the GMCs at the other 42 (52.5%) loci. Among the 42 loci with GMC decreasing alleles from the germplasms, 35 (83.3%) loci were responsible for the Cd concentration. However, among the 38 loci with GMC increasing alleles from the germplasms, 27 (71.1%) loci were responsible for the Cd concentration. Thus, according to the effects of GMC traits for human health, there were only 46 (57.5%) loci with favorable alleles from our 698 sequenced germplasms in comparison with the reference genome.

According to their physical position, the 62 loci associated with the Cd concentration could be group into 29 QTL clusters (Table 1). Sixteen (55.2%) clusters harbored at least two loci (range, 2–5; mean = 3.6 loci/cluster). The three largest clusters were found on chromosomes 8, 11, and 12. Each of them harbored five loci for Cd concentration. Reverse allelic effects from the germplasms were detected for different loci gathered in one cluster. Among 14 (48.3%) of them, a single locus was found for each cluster.

### Haplotype analysis of the GMC candidate regions

We chose a total of 10 regions with supporting evidence from our linkage mapping for candidate gene scanning. A total of 192 coding genes with wet-lab evidence according to the RAP-DB (Ohyanagi et al., 2006) were identified in eight of the ten candidate regions (Supplementary Table 3). No significant relationship was found between the annotation information and the GMC traits; therefore, all 192 genes were submitted for further analysis. Candidate gene haplotype analysis was then carried out for these genes. Statistical comparisons between the mean values of the three major GMC traits (Fe, Zn, and Cd) were then carried out for different haplotypes of the genes in the X-set and G-set, respectively.

**Figure 3:**
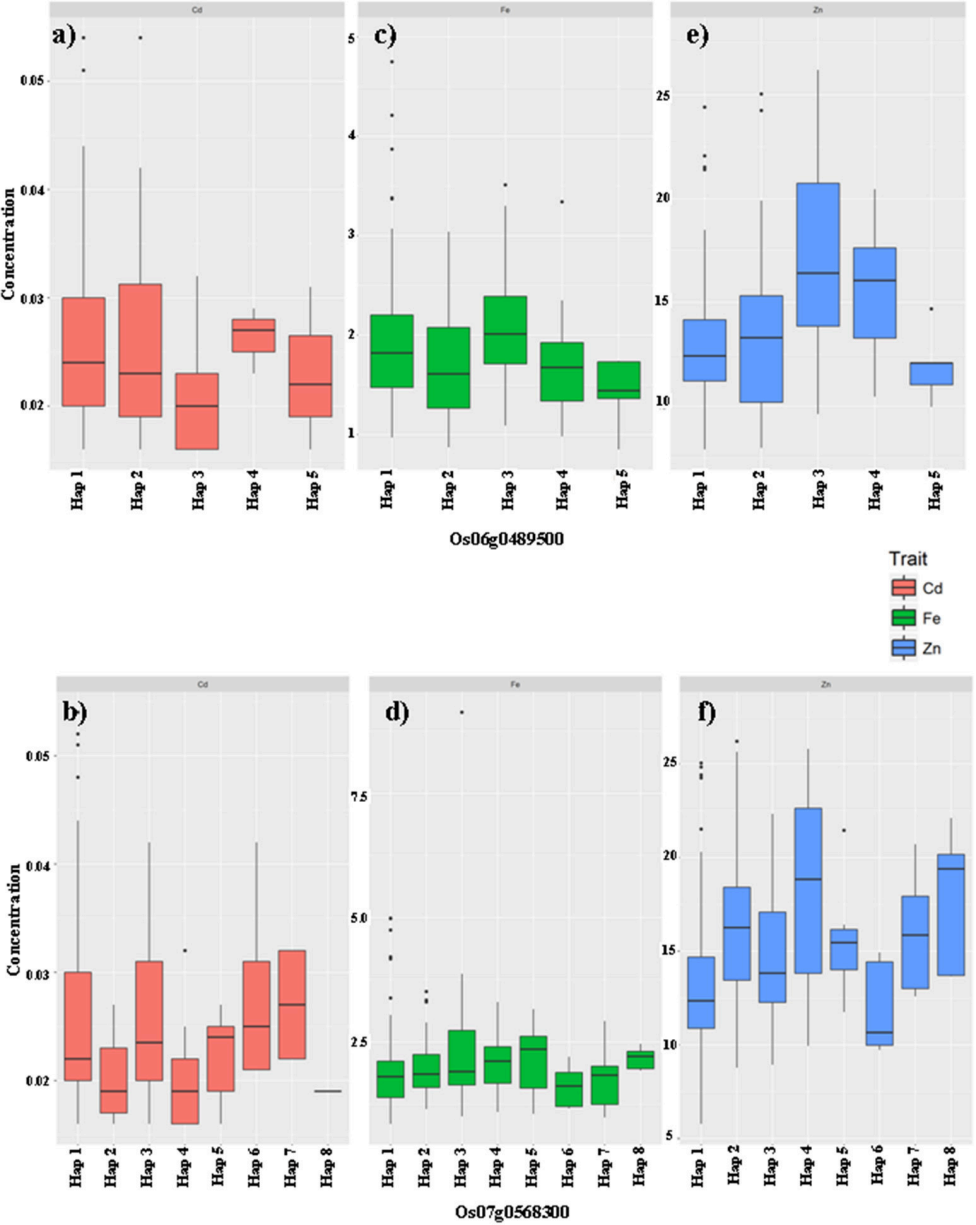
The haplotype effects of two genes Os06g0489500 **(a,c,e)**, Os07g0568300 **(b,d,f)** on the grain mineral concentration (GMC) traits (Fe: **a,b**; Zn: **c,d**; Cd: **e,f**) in milled grains of rice in germplasms from the X-set.

**Figure 4:**
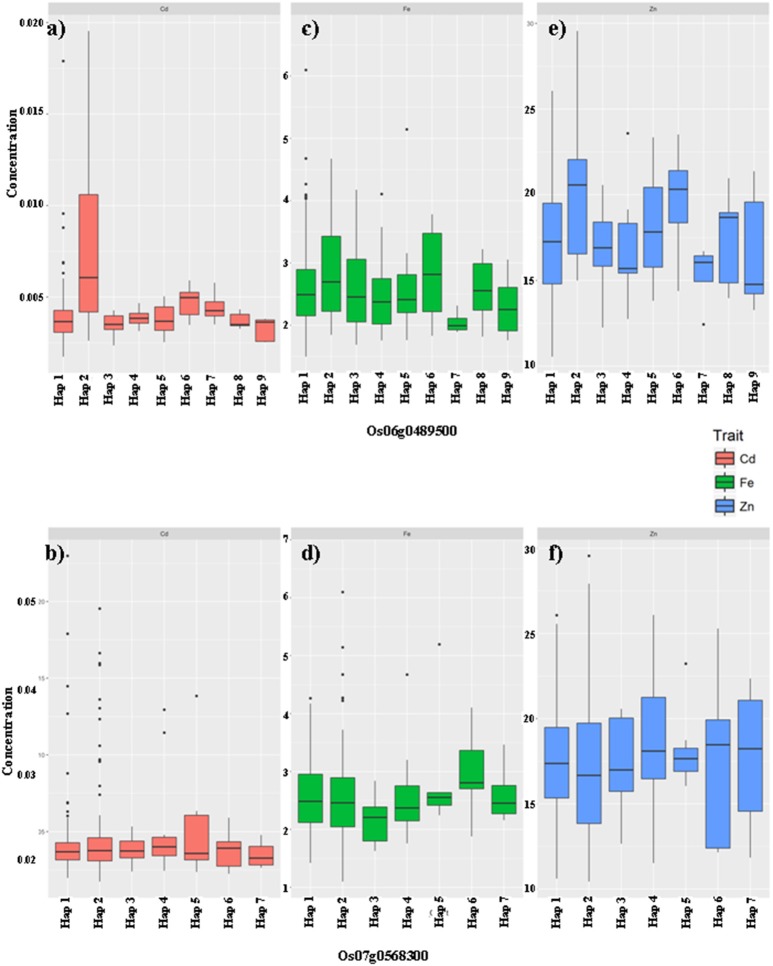
The haplotype effects of two genes Os06g0489500 **(a,c,e)**, Os07g0568300 **(b,d,f)** on the grain mineral concentration (GMC) traits (Fe: **a,b**; Zn: **c,d**; Cd: **e,f**) in milled grains of rice in germplasms from the G-set.

Based on the results of Duncan's t-test for the haplotypes of the above candidate genes, 37 genes were found to have significant associations between the haplotype variations and the targeting trait of the QTL region (Table 2). There were no obvious GMC trait-related genes based on the annotation information from RAP-DB (Supplementary Table 3); therefore, three genes associated with zinc binding domain and/or zinc finger, which have not yet been reported to be related to the GMC traits, were chosen as sample cases in addition to non-exhausted cross validations. The genes were Os06g0489500 (Chr6:16404065-17615233) for qFe6-2, and Os07g0568300 (Chr7:22841126-22941126) and Os07g0569700 (Chr7:22841126-22941126) for qZn7. We performed trait value plotting for these samples following the above tests for all 192 candidate genes. We focused on the three major GMC traits: Fe, Zn, and Cd. Most phenotypic values between the different haplotypes for Os07g0569700 were insignificant, except for the Cd concentrations in the G-set. Thus, we only showed the significant results for the other two genes [Os06g0489500 (marginally associated with Fe) and Os07g0568300 (highly associated with Zn)] in Figures 3, 4 for the X-set and G-set data, respectively.

**Table 2:** Significances of phenotypic variations among different haplotypes of 192 candidate genes within important quantitative trait locus (QTL) regions for three major grain mineral concentrations (GMCs) in milled grains in two subsets of a panel of 698 germplasms.

				**X-set^d^**			**G-set**		
**Linkage_QTL^a^**	**GWAS_Loci^b^**	**Regions**	**Candidates^c^**	**Fe**	**Zn**	**Cd**	**Fe**	**Zn**	**Cd**
qCd1	qCd1-1	Chr1:320874-353617	Os01g0106700	^*^	^****^	^***^	^*^		
	qCd1-2	Chr1:1806093-1905348	Os01g0133100	^****^	^****^	^****^			^*^
qCd4	qCd4-7	Chr4 : 30064612-30164612	Os04g0594800		^***^	^***^		^**^	^****^
			Os04g0596200		^*^			^**^	^****^
			Os04g0597300		^*^	^****^			
qCd6	qCd6-2	Chr6:10602046-10912129	Os06g0293000		^**^	^*^		^*^	^****^
			Os06g0293100	^**^	^****^	^***^	^*^	^***^	^****^
			Os06g0294100	^*^	^****^	^***^	^**^	^****^	^**^
qFe6	qFe6-2	Chr6:16404065-17615233	Os06g0483301	^****^	^****^	^***^		^*^	
			Os06g0483500	^****^	^***^	^***^		^**^	
			Os06g0483900	^****^	^****^	^***^		^**^	^*^
			Os06g0484400	^****^	^****^	^***^	^*^	^***^	
			Os06g0485100	^****^	^****^	^***^	^**^	^****^	^**^
			Os06g0486900	^**^	^**^	^**^	^**^	^**^	
			Os06g0487300	^**^	^****^	^***^	^**^	^**^	^**^
			Os06g0488200	^****^	^****^	^***^		^**^	^****^
			**Os06g0489500**	^*^	^****^	^***^		^**^	^****^
			Os06g0489900	^***^	^***^	^***^	^***^	^****^	
			Os06g0490400	^***^	^****^	^***^	^*^	^***^	^***^
			Os06g0491566	^***^	^****^	^***^	^****^	^****^	^****^
			Os06g0492300	^****^	^****^	^***^	^*^	^***^	
			Os06g0494100		^****^	^***^	^****^	^****^	^***^
			Os06g0496000	^***^	^****^	^***^	^**^	^****^	
			Os06g0496601	^****^	^**^	^*^	^**^	^****^	^****^
			Os06g0498500	^****^	^****^	^***^	^**^	^****^	^**^
			Os06g0499100	^**^	^**^		^****^	^****^	^*^
			Os06g0499500	^****^	^****^	^***^	^**^	^****^	
			Os06g0499550	^****^	^**^				
qZn7	qZn7	Chr7:22841126-22941126	**Os07g0568300**	^**^	^****^	^***^	^***^		^*^
			Os07g0568400		^****^	^***^			
			Os07g0568500		^****^	^***^	^**^	^****^	^*^
			Os07g0569000	^*^	^****^	^***^		^****^	^**^
			Os07g0569500		^**^	^*^		^**^	^****^
			Os07g0569550		^**^			^**^	^****^
			Os07g0569800		^****^	^***^	^**^	^****^	^****^
qCd8	qCd8-1	Chr8:496639-582447	Os08g0110000			^****^			
			Os08g0110600	^*^	^**^	^**^	^**^	^***^	^***^

a *QTL detected by linkage mapping in our previous report (Xu et al., 2015)*.

b *Loci detected by a genome-wide association study (GWAS) in this work*.

c *The gene highlighted as sample cases in the latter part of haplotype analysis were shown in bold*.

d *X-set = indica/Xian set, G-set = japonica / Geng set*,

*, **, ***, and **** *represents significant level of 0.05, 0.01, 0.001, and 0.0001, respectively, in the pair-wise comparison using Duncan's t-test for the different haplotypes of each gene*.

From the X-set, among the five haplotypes of Os06g0489500 (Figures 3a,c,e), Hap3 seemed to be the most favorable one, which is associated with relatively higher Fe and Zn concentrations, but without significant affects on the Cd concentration, compared with the other haplotypes. Hap1 was the second choice. It was associated with an increased Fe concentration, but a relatively lower Zn concentration, and an insignificantly higher Cd concentration. Among the eight haplotypes of Os07g0568300 (Figures 3b,d,f), Hap6 seemed to be the most unfavorable one, being associated with relatively lower Fe and Zn concentrations, and an insignificantly higher Cd concentration. Hap4 and Hap8 from the X-set were only associated with higher Zn concentration and had no significant effect on the Fe or Cd concentrations. Additionally, mild but significant effects of Hap2, Hap5, and Hap7 on Zn concentrations were also detected compared with Hap6.

In the G-set (Figure 4), among the nine haplotypes of Os06g0489500, Hap2 increased not only Zn but also Cd concentrations compared with the other haplotypes. Hap7 significantly reduced Zn, but had insignificantly increased the Cd concentration. Os07g0568300 was only associated with the Fe concentrations in the G-set. Among the seven haplotypes, Hap6 was favorable, which significantly increased the Fe, but had no significant effects on the Zn or Cd concentrations.

In addition to these two significant candidate genes shown in sample cases, all 37 genes listed in Table 2 will become the focus for further functional verification in our future work.

## Discussion

### Comparison of identified GMC QTL with reported genes/QTL

As described in another report for GMC QTL mapping in milled grain of rice (Hu et al., 2016), the statistical significances of QTL for the GMCs in milled grain are much lower compared with the QTL detected for GMCs in brown rice grains. This phenomenon also appeared in our association mapping experiment. Thus, we adopted two thresholds, including a relative loose one to minimize the type II error. Finally, we mapped a total of 80 loci (Table 1, Figure 2). Ten (12.5%) of them including *qFe6-2, qFe7, qZn7, qZn12, qCd1-1*/*qCd1-2, qCd4-7, qCd6-2, qCd8-1*, and *qCd11-1* were consistent with the loci from our previous linkage mapping work, including *qFe6, qFe7, qZn7, qZn12, qCd1, qCd4, qCd6, qCd8*, and *qCd11*, respectively.

Twenty (25%) of these 80 loci were also supported by loci reported in other works. Some were supported by multiple references. For example, *qZn1* covered the region marked by id1005056–id1005058 (Norton et al., 2014), *qZn7* was consistent with *qZn7* (Huang et al., 2015; Hu et al., 2016) and *qZN-7* (Lu et al., 2008), as well as the marker id7003641, which was significantly associated with the Zn concentration (Norton et al., 2014). QTL *qCd7-1* was supported by *qSCd7/ qGCd7* (Ishikawa et al., 2010) and *qCdp7* (Abe et al., 2011). Some QTL were supported by single piece of evidence. The QTL *qCd2-2* was consistent with *qCd2b* (Zhang et al., 2014). The loci *qCd3-1*, and *qCd3-5* were consistent with two different reported *qCd3* (Zhang et al., 2014; Huang et al., 2015), while the loci *qCd11-8*, and *qCd11-9* were covered by a same relatively large region of *qCd11* (Kashiwagi et al., 2009). The other 12 loci, *qCd4-7, qCd5-1, qCd5-7, qCd6-2, qCd6-3, qCd6-6, qCd7-2, qCd8-1, qCd8-3, qCd11-7, qCd12-9*, and *qCu5* were consistent with *qCd4-2* (Kashiwagi et al., 2009), *qCd5* (Zhang et al., 2014), *qCd5.1* (Huang et al., 2015), Segment_on_Chr6 (Ishikawa et al., 2005), *OsLCT1* (Uraguchi et al., 2014), *qCd6* (Zhang et al., 2014), *qCDCN-7* (Shen et al., 2008), Segment_on_Chr8(Ishikawa et al., 2005), *qCd8* (Zhang et al., 2014), *qCd11* (Tang, 2007), *qSCd12* (Ishikawa et al., 2010), and *qCu5* (Zhang et al., 2014), respectively.

Notably, five loci (6.3%) including *qZn7, qCd4-7, qCd6-2*, and *qCd8-1* were supported by multiple pieces of evidence from our linkage mapping and other references. Thus, they would be of higher value for breeding application, with characteristics of stable expression and/or genetic background independence.

### Multiple evidence for QTL detection for GMCs

Although, the statistical significance for *qFe6-2* and *qZn7* was only marginal (both with –log_10_ = 3.6) in GWAS mapping, they still possessed independent supporting evidence from the linkage mapping work (Xu et al., 2015). Additionally, there were supporting references of *qZn7* (Huang et al., 2015; Hu et al., 2016), and *qZN-7* (Lu et al., 2008), and the significant marker regions of id1005056–id1005058 (Norton et al., 2014). Thus, the joint application of the GWAS and linkage mapping again showed its power for QTL mapping, even for the traits with relatively low heritability, such as the GMC traits. Sometimes, multiple independent marginal evidences, when taken together, are more powerful than one single strong association signal.

*Japonica*/*Geng* and *indica*/*Xian* differ markedly in their ability to accumulate Cd (Ueno et al., 2010; Uraguchi et al., 2011), which is much more significant than for the other GMC traits. Thus, when we pooled the two subsets together for the analysis, a population similar to those used for bulk-segregant analysis, with a bi-nominal distribution, was formed. This explained why the Cd QTL gained more statistical power in the GWAS mapping (Figures 1C, 2C). By contrast, the distributions of other GMC traits were not so significantly associated with the population structure, when divided by subsets (Figures 1A,B,D–F). In addition, only the locus significant in both sets, or at least highly significantly in one set, would be detected within the pooled data. Those peaks with an average level of significance in a single set would be highly likely to decrease in the analyses using the pooled data. However, this kind of underestimation of the QTL underlying the other five GMC traits would not have a large affect on the exploration of the really important loci that are suitable for practical breeding, especially those with multiple pieces of evidence that support the QTL, such as *qZn7* and *qFe6-2*.

Additionally, many closely linked QTL with reverse allelic effects for the Cd concentration were identified in QTL clusters along all the chromosomes, except for chromosomes 2 and 7. Thirteen (44.8%) of these clusters were supported by evidence from our previous mapping work or by other reports (Table 2). The largest clusters, Clst8C, Clst11b, and Clst12b, were each was found to harbor five loci for Cd concentration. Clst11b was also supported by evidence from multiple references. In our previous report, genetic overlaps were found for QTL controlling different GMC traits. Commonly, chromosomal crossovers in this kind of germplasm panel were thought to occur more frequently than in a bi-parental population. Thus, in this mapping work, with the improvement of mapping resolution compared with SSR linkage mapping in a bi-parental population, the details of the genetic overlap between GMC traits, especially those caused by tight linkage, may be magnified. The exact mechanisms underlying these Cd regions require further investigation.

Finally, according to the joint favorable haplotype exploration, we found that functional annotation could not always offer sufficient useful information during the candidate genes screening. By contrast, the QTL targeting trait comparison would effectively help to narrow down the candidate genes from 192 to 37 by removing more than 80% unrelated information.

### Implications for molecular biofortification breeding

This work offers at least three useful implications for the biofortification molecular breeding of rice. The first is that the QTL or candidate gene haplotypes underlying the GMC traits detected in this report, as well as those from our previous report (Xu et al., 2015), showed multiple effects on more than one GMC trait. Thus, in biofortification molecular breeding work on crops, especially rice (*Oryza sativa* L.), a possible trade-off between the improvement of favorable GMCs, such as Fe and Zn, and the accumulation of toxic heavy metal elements, such as Cd, in the milled grain should be taken into consideration. Selection of favorable haplotypes of candidate genes during molecular breeding would decide the final success of the breeding products. For example, if we chose Hap3 from the X-set for Os06g0489500, a relatively higher Fe and Zn concentration in the milled grain would be obtained, together with an insignificantly lower Cd concentration; however, if Hap1 of the X-set was adopted, the improved Fe concentration would be accompanied by a relatively lower Zn concentration and an insignificantly higher Cd concentration (Figures 3a,c,e). Thus, when we construct a scheme for backcross (BC) breeding, which is commonly adopted in biofortification breeding, using certain germplasms with higher favorable GMCs, such as Fe and/or Zn, as donors and an elite line as recurrent parents (RPs), at least two important steps should be taken during parental selection. First, the existing haplotypes of the target genes in the RPs and donors should be clarified by genotyping and haplotype analysis. Second, different GMCs, especially nutrient minerals and toxic minerals, should be balanced. For different RPs, different elite donors with suitable haplotypes should be selected for crossing.

The second point is that according to the mean values for the GMCs based on the haplotypes in the X-set and G-set, the Cd concentration is significantly lower in the G-set. This is consistent with known differences in Cd accumulation between *indica*/*Xian* and *japonica*/*Geng* (Ueno et al., 2010; Uraguchi et al., 2011). Thus, not only could the favorable haplotypes within the subspecies be used, but also those from across the subspecies could be taken into consideration. For example, for hybrid breeding, where most products belong to *indica*/*Xian* type, favorable haplotypes to decrease the unfavorable GMCs, such as Cd, could be imported from the *japonica* /*Geng* donors.

Finally, by combining the joint exploration of the GWAS mapping results with the results from our previous linkage mapping work, and the reference data from other reports, it was possible to identify the QTL regions for the GMCs in the milled grain more reliably. All the mapped loci, especially those that were jointly detected, as well as their favorable haplotypes, offer an opportunity to enhance the Fe and/or Zn concentrations, but control Cd accumulation, in milled rice grains. Biofortification molecular breeding using the favorable haplotypes jointly explored in this work, involving marker assisted selection and/or gene editing, would be the next step of our on-going studies.

## Author contributions

T-QZ, J-LX, and Z-KL: Conceived and designed the experiments; T-QZ, G-MZ, Y-MS, Y-LW, and YW: Performed the experiments; C-CW and T-QZ: Analyzed the data; ZC, C-ZL, T-TX, L-YZ, J-TM, L-WD, and WL: Contributed reagents, materials, and analysis tools; T-QZ and J-LX: Wrote the paper.

### Conflict of interest statement

The authors declare that the research was conducted in the absence of any commercial or financial relationships that could be construed as a potential conflict of interest. The reviewer QS and handling Editor declared their shared affiliation.

## Supplementary material

Supplementary Figure 1 Comparison of populations presented by clustering and principal component analysis (PCA) in three sets of data.

Supplementary Figure 2 Comparison of genome-wide association study (GWAS) mapping results in three sets of data for Fe in the milled grains.

Supplementary Figure 3 Comparison of genome-wide association study (GWAS) mapping results in three sets of data for Zn in the milled grains.

Supplementary Figure 4 Comparison of genome-wide association study (GWAS) mapping results in three sets of data for Cd in the milled grains.

Supplementary Figure 5 Comparison of genome-wide association study (GWAS) mapping results in three sets of data for Mn in the milled grains.

Supplementary Figure 6 Comparison of genome-wide association study (GWAS) mapping results in three sets of data for Cu in the milled grains.

Supplementary Figure 7 Comparison of genome-wide association study (GWAS) mapping results in three sets of data for Se in the milled grains.

Supplementary Table 1 Physical and chemical characteristics of the soil in the experimental fields.

Supplementary Table 2 Analysis of variance (ANOVA) for the control panel under two environments.

Supplementary Table 3 Coding genes in the candidate regions used for haplotype analysis.
