# Supplementary figures and images for: Joint Exploration of Favorable Haplotypes for Mineral Concentrations in Milled Grains of Rice (Oryza sativa L.)
Source: Front Plant Sci. 2018 Apr 12;9:447. doi: 10.3389/fpls.2018.00447 (PMC5906679; doi:10.3389/fpls.2018.00447)

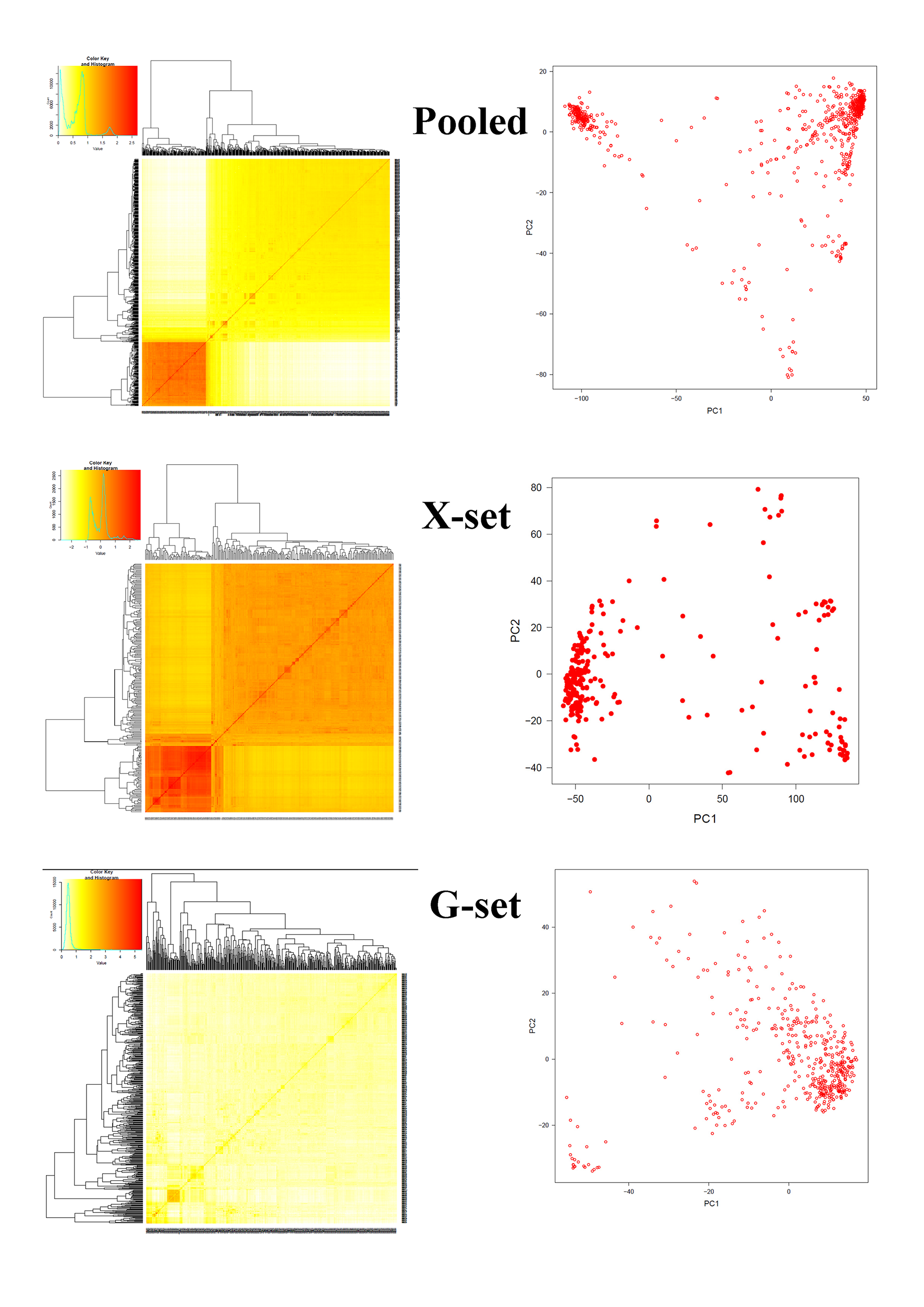

Supplement: Supplementary Figure 1 — Comparison of populations presented by clustering and principal component analysis (PCA) in three sets of data. [file Image1.TIF]

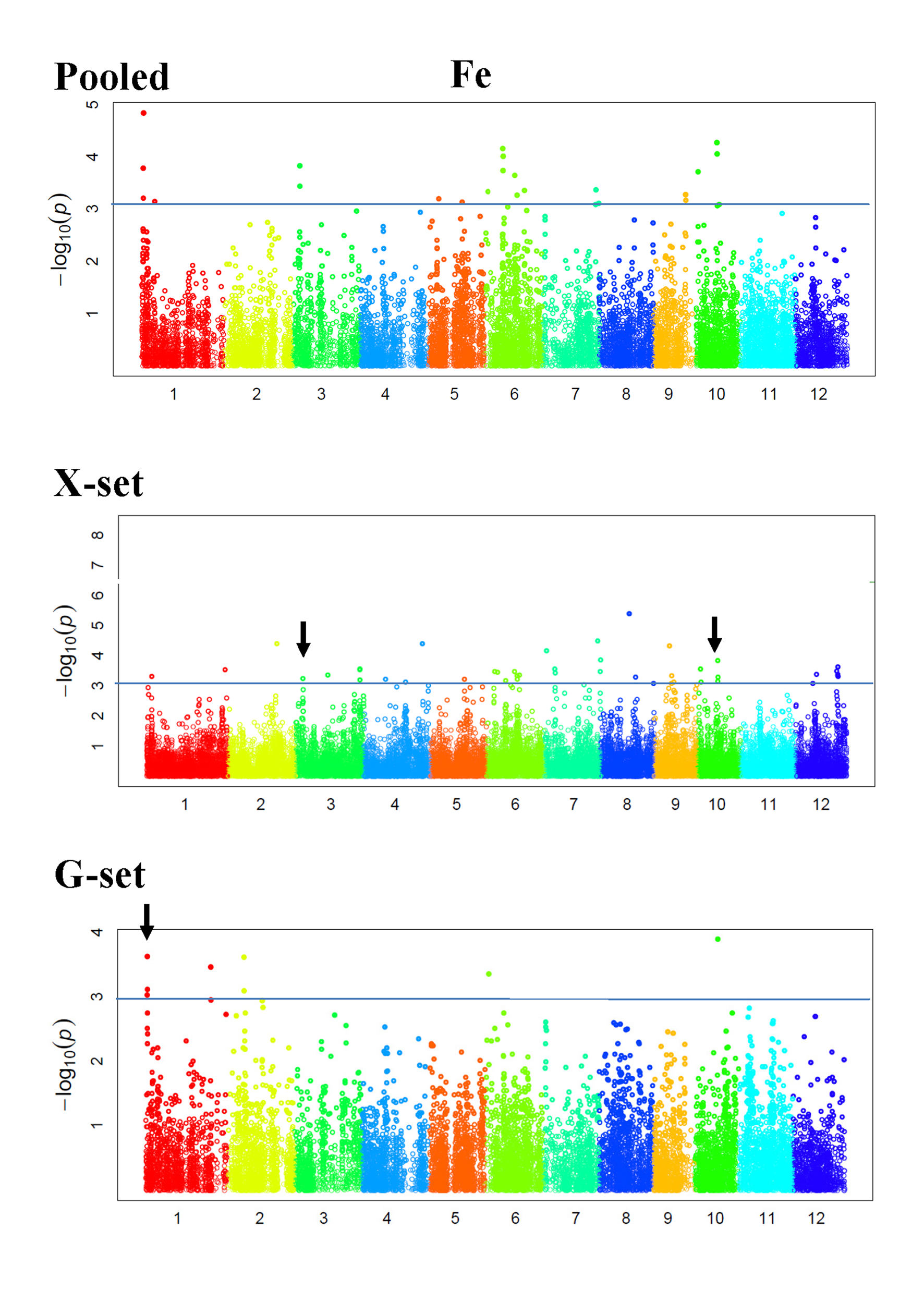

Supplement: Supplementary Figure 2 — Comparison of genome-wide association study (GWAS) mapping results in three sets of data for Fe in the milled grains. [file Image2.TIF]

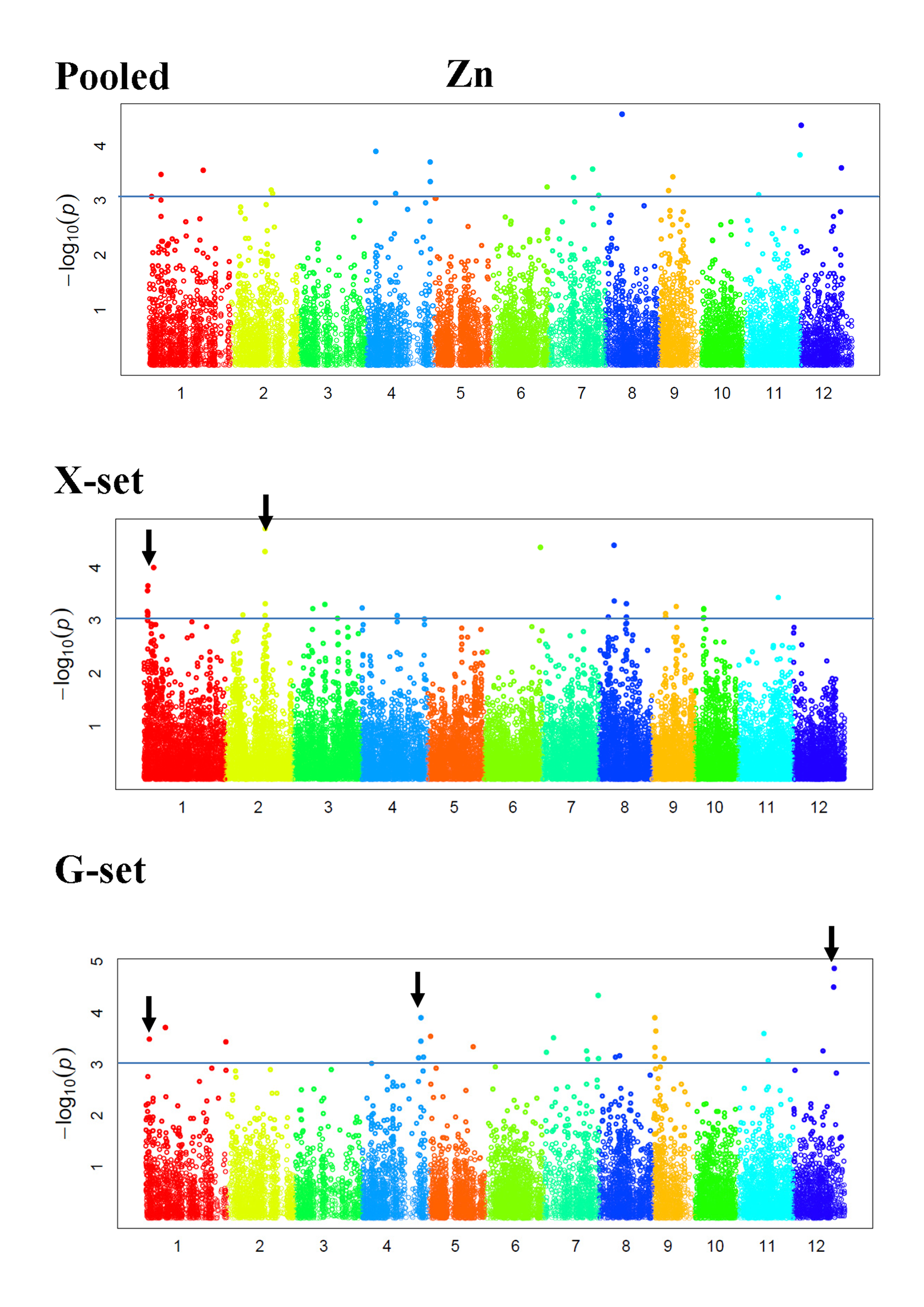

Supplement: Supplementary Figure 3 — Comparison of genome-wide association study (GWAS) mapping results in three sets of data for Zn in the milled grains. [file Image3.TIF]

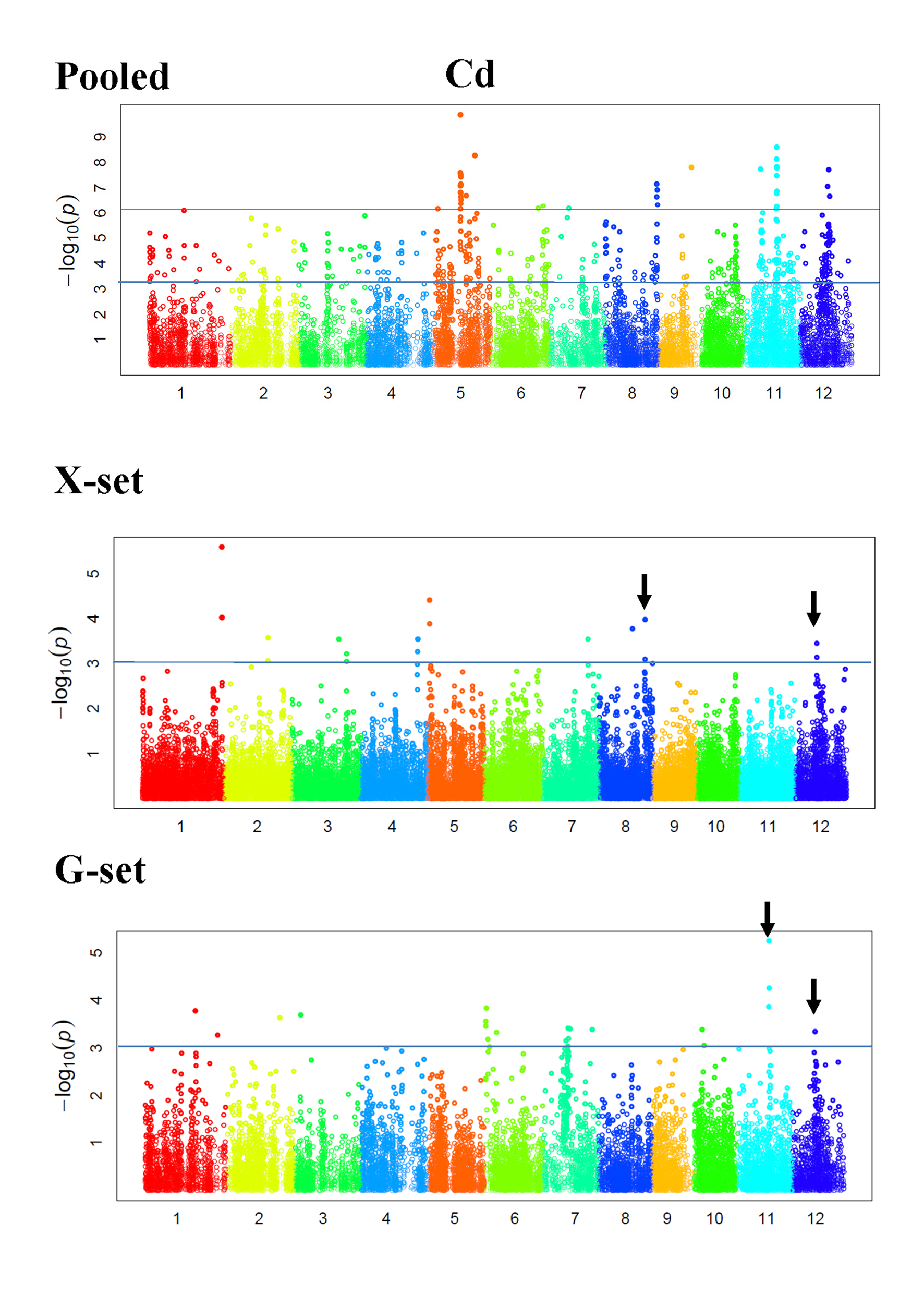

Supplement: Supplementary Figure 4 — Comparison of genome-wide association study (GWAS) mapping results in three sets of data for Cd in the milled grains. [file Image4.TIF]

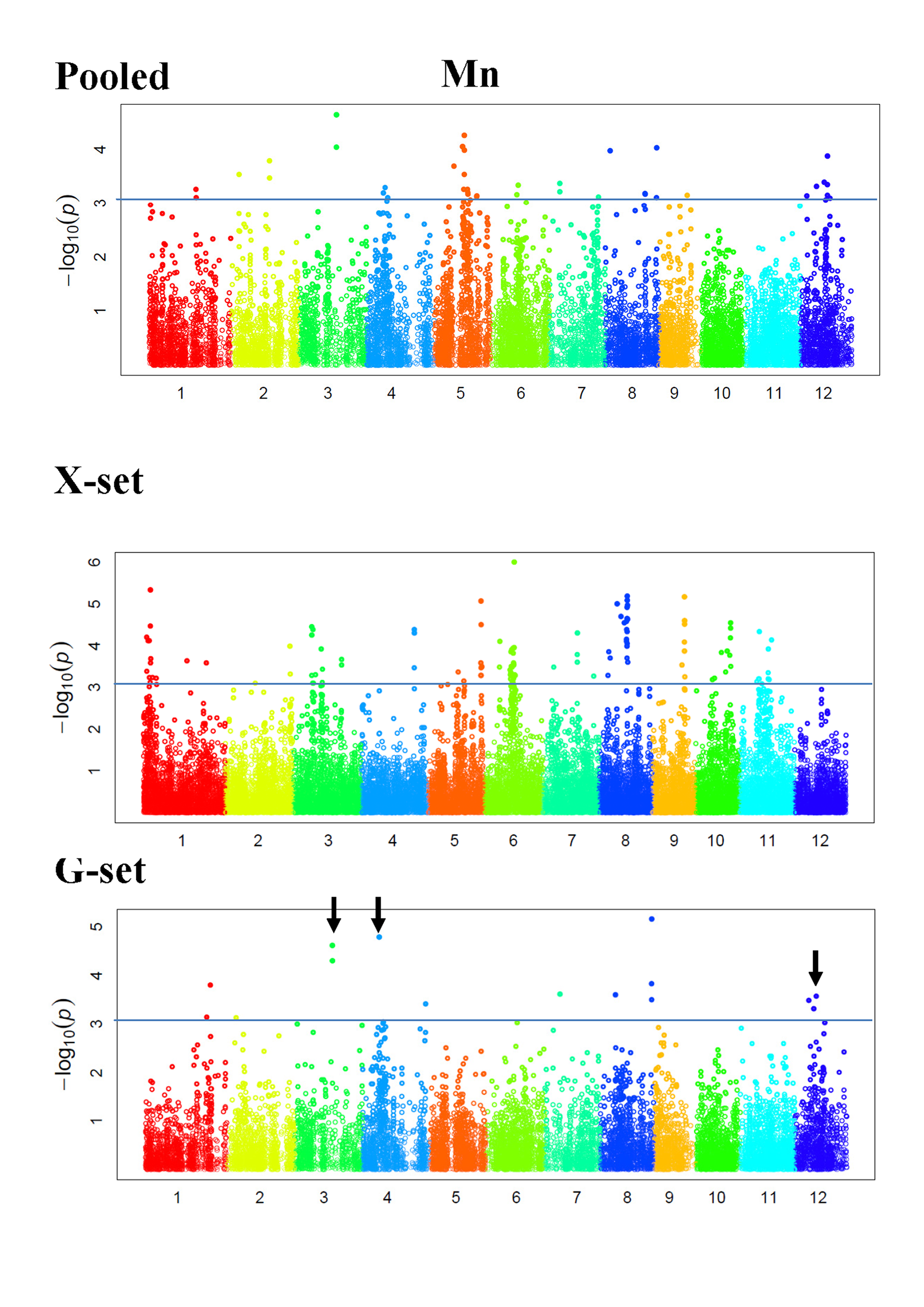

Supplement: Supplementary Figure 5 — Comparison of genome-wide association study (GWAS) mapping results in three sets of data for Mn in the milled grains. [file Image5.TIF]

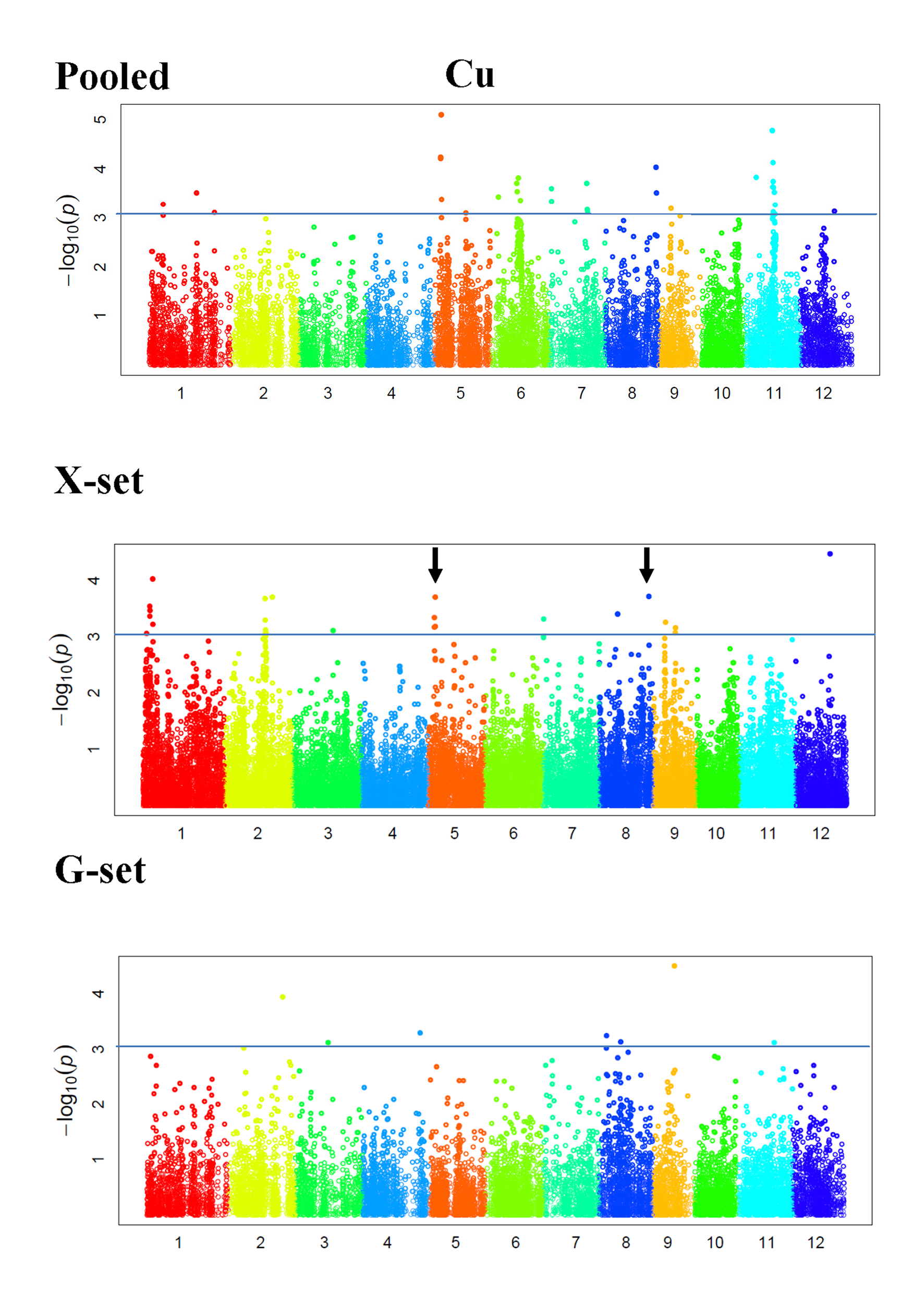

Supplement: Supplementary Figure 6 — Comparison of genome-wide association study (GWAS) mapping results in three sets of data for Cu in the milled grains. [file Image6.TIF]

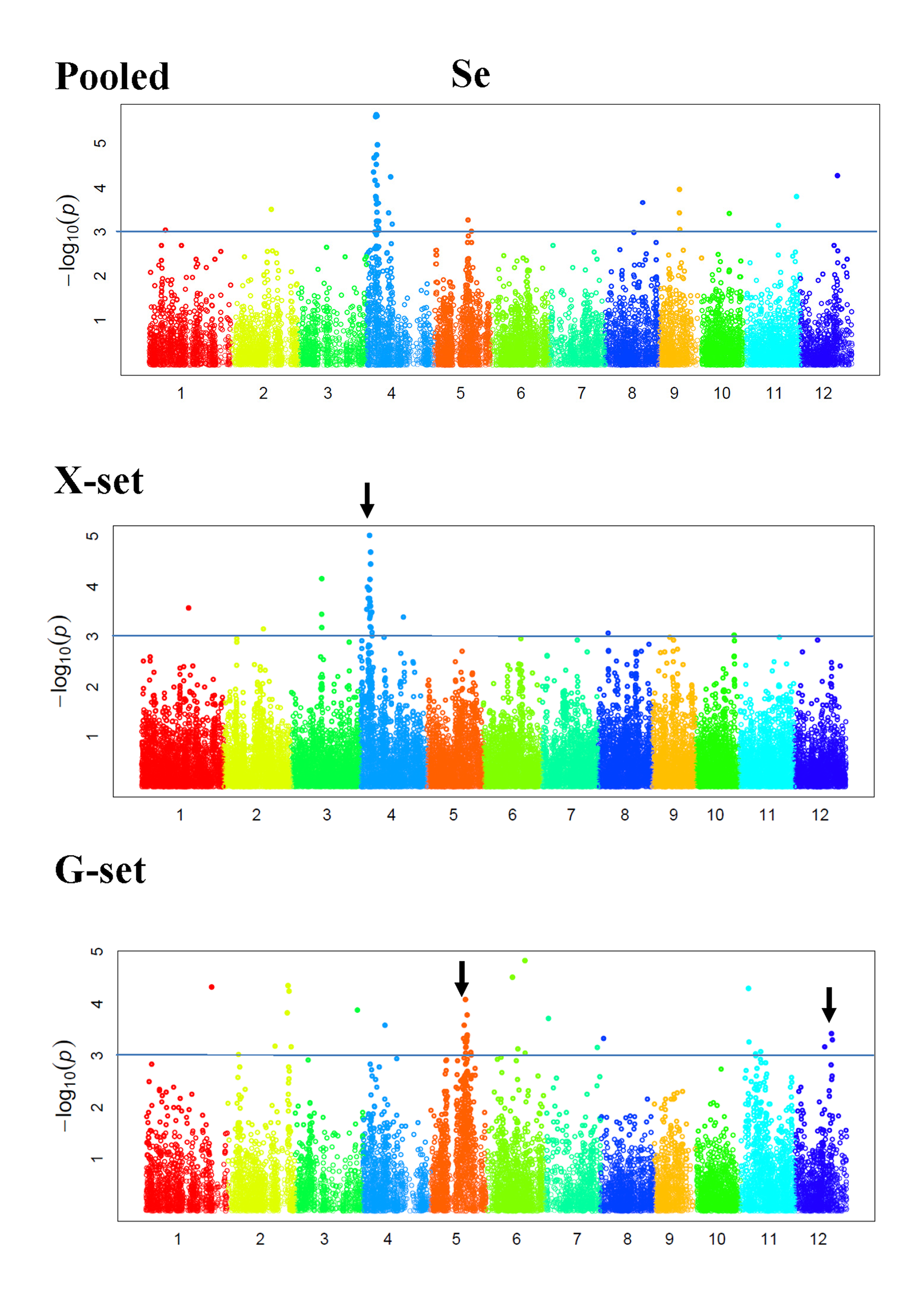

Supplement: Supplementary Figure 7 — Comparison of genome-wide association study (GWAS) mapping results in three sets of data for Se in the milled grains. [file Image7.TIF]
