# Supplementary material for: Joint Exploration of Favorable Haplotypes for Mineral Concentrations in Milled Grains of Rice (Oryza sativa L.)
Source: Front Plant Sci. 2018 Apr 12;9:447. doi: 10.3389/fpls.2018.00447 (PMC5906679; doi:10.3389/fpls.2018.00447)
Supplement: Supplementary Table 1 — Physical and chemical characteristics of the soil in the experimental fields. [file Table1.doc]

**Supplementary Table 1. Physical and chemical characteristics of the soil in the experimental fields**

| **Item** | **Sanya** | **Haerbin** |
| --- | --- | --- |
| pH | 6.81 | 6.00 |
| Organic matter (g kg^-1^) | 20.16 | 31.50 |
| Total N (g kg^-1^) | 7.10 | 25.25 |
| Total P (g kg^-1^) | 0.36 | 18.55 |
| Total K (g kg^-1^) | 28.15 | 34.39 |
| Available N(mg kg^-1^) | 93.81 | 209.62 |
| Available P(mg kg^-1^) | 19.47 | 15.41 |
| Available K(mg kg^-1^) | 70.73 | 173.34 |
| Total Fe (mg kg^-1^) | 1992.60 | 2176.53 |
| Total Zn (mg kg^-1^) | 1107.31 | 1292.32 |
| Total Cd (mg kg^-1^) | 0.27 | 0.22 |
| Total Mn (mg kg^-1^) | 1687.50 | 2411.54 |
| Total Cu (mg kg^-1^) | 36.86 | 37.26 |
| Total Se (mg kg^-1^) | 0.32 | 0.21 |
| Available Fe (mg kg^-1^) | 4.33 | 8.75 |
| Available Zn (mg kg^-1^) | 24.04 | 31.85 |
| Available Cd (mg kg^-1^) | 0.13 | 0.12 |
| Available Mn (mg kg^-1^) | 366.30 | 594.30 |
| Available Cu (mg kg^-1^) | 18.29 | 20.00 |
| Available Se (mg kg^-1^) | 0.04 | 0.03 |
