# Supplementary material for: Joint Exploration of Favorable Haplotypes for Mineral Concentrations in Milled Grains of Rice (Oryza sativa L.)
Source: Front Plant Sci. 2018 Apr 12;9:447. doi: 10.3389/fpls.2018.00447 (PMC5906679; doi:10.3389/fpls.2018.00447)
Supplement: Supplementary Table 2 — Analysis of variance (ANOVA) for the control panel under two environments. [file Table2.doc]

**Supplementary Table 2. Analysis of variance (ANOVA) for the control panel under two environments.**

| **Trait** | **Source ^1^** | **DF** | **SS** | **MS** | **F** | **Pr > F** |
| --- | --- | --- | --- | --- | --- | --- |
| Fe | Geno | 6 | 3.14456 | 0.524093 | 2.334 | 0.1630 |
|  | Env | 1 | 0.008732 | 0.008732 | 0.039 | 0.8502 |
|  | Error | 6 | 1.347224 | 0.224537 |  |  |
| Zn | Geno | 6 | 104.7267 | 17.45445 | 5.263 | 0.0316 |
|  | Env | 1 | 4.883234 | 4.883234 | 1.472 | 0.2706 |
|  | Error | 6 | 19.89894 | 3.31649 |  |  |
| Cd | Geno | 6 | 0.000177 | 2.95E-05 | 2.342 | 0.1621 |
|  | Env | 1 | 1.1E-07 | 1.1E-07 | 0.009 | 0.9290 |
|  | Error | 6 | 7.57E-05 | 1.26E-05 |  |  |
| Mn | Geno | 6 | 8.10E-06 | 1.35E-06 | 6.749 | 0.0185 |
|  | Env | 1 | 2.33E-06 | 2.33E-06 | 11.650 | 0.0149 |
|  | Error | 6 | 1.23E-06 | 2E-07 |  |  |
| Cu | Geno | 6 | 22.88145 | 3.813575 | 3.371 | 0.0824 |
|  | Env | 1 | 0.023254 | 0.023254 | 0.021 | 0.8907 |
|  | Error | 6 | 6.787631 | 1.131272 |  |  |
| Se | Geno | 6 | 3.707817 | 0.61797 | 1.930 | 0.2218 |
|  | Env | 1 | 0.00079 | 0.00079 | 0.002 | 0.9620 |
|  | Error | 6 | 1.920855 | 0.320143 |  |  |

^1^ Geno = Genotypic factors; Env = Evironmental factors; Error = Random errors; DF = Degree of freedom; SS = Sum of square; MS = Mean value of SS.
