# Supplementary material for: Joint Exploration of Favorable Haplotypes for Mineral Concentrations in Milled Grains of Rice (Oryza sativa L.)
Source: Front Plant Sci. 2018 Apr 12;9:447. doi: 10.3389/fpls.2018.00447 (PMC5906679; doi:10.3389/fpls.2018.00447)
Supplement: Supplementary Table 3 — Coding genes in the candidate regions used for haplotype analysis. [file Table3.doc]

**Supplementary Table 3. Coding genes in the candidate regions used for haplotype analysis.**

| **Region** | **Candidates** | **Strain** | **Function** | **Notes** |
| --- | --- | --- | --- | --- |
| Chr1:320874-353617 |  |  |  |  |
|  | Os01g0106200 | + | Similar to RER1A protein (AtRER1A). (Os01t0106200-01) |  |
|  | Os01g0106300 | - | Similar to Isoflavone reductase homolog IRL (EC 1.3.1.-). (Os01t0106300-01) |  |
|  | Os01g0106400 | - | Similar to Isoflavone reductase homolog IRL (EC 1.3.1.-). (Os01t0106400-01) |  |
|  | Os01g0106500 | - | Conserved hypothetical protein. (Os01t0106500-01) |  |
|  | Os01g0106600 | - | Similar to Chitin-binding lectin 1 precursor (PL-I). (Os01t0106600-01) |  |
|  | Os01g0106700 | + | Hypothetical conserved gene. (Os01t0106700-00) | ATM homolog |
| Chr1:1806093-1905348 |  |  |  |  |
|  | Os01g0132800 | - | Peptidyl-tRNA hydrolase family protein. (Os01t0132800-01) |  |
|  | Os01g0133100 | + | Conserved hypothetical protein. (Os01t0133100-01)%3BHypothetical conserved gene. (Os01t0133100-02) |  |
|  | Os01g0133200 | - | Conserved hypothetical protein. (Os01t0133200-01) |  |
|  | Os01g0133400 | + | Similar to Hexose transporter (Fragment). (Os01t0133400-01) | PGLCT, PLASTIDIC GLUCOSE TRANSLOCATOR, OsPGLCT |
|  | Os01g0133500 | + | Beta-lactamase-like domain containing protein. (Os01t0133500-01)%3BSimilar to predicted protein. (Os01t0133500-02) |  |
|  | Os01g0133600 | + | Hypothetical conserved gene. (Os01t0133600-00) |  |
|  | Os01g0133650 | - | Hypothetical gene. (Os01t0133650-00) |  |
|  | Os01g0133700 | + | Glycosyl transferase family 14 domain containing protein. (Os01t0133700-00) |  |
|  | Os01g0133766 | - | Non-protein coding transcript. (Os01t0133766-00) |  |
|  | Os01g0133799 | - | Hypothetical protein. (Os01t0133799-00) |  |
|  | Os01g0133832 | - | Hypothetical gene. (Os01t0133832-00) |  |
|  | Os01g0133866 | - | Hypothetical protein. (Os01t0133866-00) |  |
|  | Os01g0133900 | + | Similar to predicted protein. (Os01t0133900-00) |  |
|  | Os01g0134200 | + | Similar to Universal stress protein family protein. (Os01t0134200-01)%3BHypothetical conserved gene. (Os01t0134200-02) |  |
|  | Os01g0134500 | + | Similar to Delta-7-sterol-C5(6)-desaturase (EC 1.3.3.-) (Delta-7-C-5 sterol desaturase) (Delta7-sterol-C5-desaturase). (Os01t0134500-01) | OsSTE1 OsDWF7, Sterol methyltransferase 1 Dwarf 7 |
|  | Os01g0134700 | + | Calmodulin binding protein-like family protein. (Os01t0134700-01) | Calmodulin binding protein |
| Chr4:30064612-30164612 |  |  |  |  |
|  | Os04g0594800 | + | Similar to OSIGBa0142I02-OSIGBa0101B20.31 protein. (Os04t0594800-01) | OsOPT6, Oligopeptide transporter 6 |
|  | Os04g0595125 | - | Hypothetical protein. (Os04t0595125-00) |  |
|  | Os04g0595450 | - | Hypothetical conserved gene. (Os04t0595450-00) |  |
|  | Os04g0595583 | - | Conserved hypothetical protein. (Os04t0595583-00) |  |
|  | Os04g0596200 | + | Peptidase A1 domain containing protein. (Os04t0596200-01) |  |
|  | Os04g0596300 | - | Protein of unknown function DUF760 family protein. (Os04t0596300-01)%3BSimilar to predicted protein. (Os04t0596300-02) |  |
|  | Os04g0596350 | + | Non-protein coding transcript. (Os04t0596350-00) |  |
|  | Os04g0596400 | - | Similar to UPF0195 protein CG30152. (Os04t0596400-01) |  |
|  | Os04g0596500 | - | Non-protein coding transcript. (Os04t0596500-01)%3BTranslation elongation factor EFTu/EF1A C-terminal domain containing protein. (Os04t0596500-02) |  |
|  | Os04g0596900 | - | Phosphoribulokinase/uridine kinase family protein. (Os04t0596900-00) |  |
|  | Os04g0597000 | - | Similar to Secretory carrier membrane protein. (Os04t0597000-01)%3BSimilar to Secretory carrier membrane protein. (Os04t0597000-02) |  |
|  | Os04g0597300 | + | Similar to WERKY protein (Fragment). (Os04t0597300-01) | WRKY37, WRKY GENE 37, OsWRKY37, Rice WRKY gene37 |
|  | Os04g0597400 | + | Similar to OSJNba0093F12.10 protein. (Os04t0597400-00) |  |
|  | Os04g0597501 | + | Similar to OSJNba0093F12.10 protein. (Os04t0597501-00) |  |
|  | Os04g0597534 | - | Hypothetical protein. (Os04t0597534-00) |  |
|  | Os04g0597600 | + | TGF-beta receptor type I/II extracellular region family protein. (Os04t0597600-01) | PTR5, PROTEIN TRANSPORTER 5, OsPTR5 |
|  | Os04g0597700 | - | Hypothetical protein. (Os04t0597700-01) |  |
|  | Os04g0597800 | + | TGF-beta receptor type I/II extracellular region family protein. (Os04t0597800-01) | PTR6, PROTEIN TRANSPORTER 6, OsPTR6 OsNRT1%3B2, PTR/NRT1 transporter 6 |
|  | Os04g0597816 | - | Hypothetical protein. (Os04t0597816-00) |  |
| Chr6:10602046-10912129 |  |  |  |  |
|  | Os06g0290701 | - | Hypothetical protein. (Os06t0290701-00) |  |
|  | Os06g0291100 | + | Non-protein coding transcript. (Os06t0291100-00) |  |
|  | Os06g0291500 | + | Conserved hypothetical protein. (Os06t0291500-01) |  |
|  | Os06g0291600 | - | Similar to Protein kinase G11A (EC 2.7.1.-) (Fragment). (Os06t0291600-01) | G11A |
|  | Os06g0291800 | - | Conserved hypothetical protein. (Os06t0291800-00) |  |
|  | Os06g0292100 | - | Hypothetical conserved gene. (Os06t0292100-00) |  |
|  | Os06g0292301 | + | Hypothetical gene. (Os06t0292301-01) |  |
|  | Os06g0292400 | - | Embryogenesis transmembrane protein. (Os06t0292400-00) |  |
|  | Os06g0292600 | + | Non-protein coding transcript. (Os06t0292600-01) |  |
|  | Os06g0293000 | + | Hypothetical conserved gene. (Os06t0293000-00) |  |
|  | Os06g0293050 | - | Hypothetical protein. (Os06t0293050-00) |  |
|  | Os06g0293100 | + | Conserved hypothetical protein. (Os06t0293100-00) |  |
|  | Os06g0293300 | - | Hypothetical protein. (Os06t0293300-00) |  |
|  | Os06g0293500 | - | Conserved hypothetical protein. (Os06t0293500-01) |  |
|  | Os06g0293583 | + | Hypothetical protein. (Os06t0293583-00) |  |
|  | Os06g0294000 | - | Conserved hypothetical protein. (Os06t0294000-01) |  |
|  | Os06g0294100 | + | Hypothetical protein. (Os06t0294100-00) |  |
|  | Os06g0294200 | - | Conserved hypothetical protein. (Os06t0294200-01) |  |
|  | Os06g0294300 | + | Hypothetical protein. (Os06t0294300-00) |  |
|  | Os06g0294400 | - | Conserved hypothetical protein. (Os06t0294400-00) |  |
|  | Os06g0294501 | - | Non-protein coding transcript. (Os06t0294501-01) |  |
|  | Os06g0294600 | - | Cytochrome P450 family protein. (Os06t0294600-02) |  |
|  | Os06g0294775 | + | Hypothetical protein. (Os06t0294775-00) |  |
|  | Os06g0294950 | - | Conserved hypothetical protein. (Os06t0294950-01) |  |
|  | Os06g0295000 | - | Hypothetical conserved gene. (Os06t0295000-01) |  |
|  | Os06g0295250 | - | Non-protein coding transcript. (Os06t0295250-00) |  |
|  | Os06g0295300 | + | Conserved hypothetical protein. (Os06t0295300-00) |  |
|  | Os06g0295366 | - | Hypothetical gene. (Os06t0295366-01) |  |
|  | Os06g0295432 | - | Non-protein coding transcript. (Os06t0295432-00) |  |
|  | Os06g0295500 | - | Hypothetical gene. (Os06t0295500-01)%3BHypothetical protein. (Os06t0295500-02)%3BNon-protein coding transcript. (Os06t0295500-03) |  |
| Chr6:16404065-17615233 |  |  |  |  |
|  | Os06g0483200 | - | Similar to cycloartenol synthase. (Os06t0483200-01)%3BSimilar to Beta-amyrin synthase. (Os06t0483200-02) | OsOSC6, oxidosqualene cyclase 6 |
|  | Os06g0483301 | - | Hypothetical gene. (Os06t0483301-01) |  |
|  | Os06g0483500 | - | Similar to H0124B04.15 protein. (Os06t0483500-01)%3BSimilar to H0124B04.15 protein. (Os06t0483500-02) |  |
|  | Os06g0483701 | - | Hypothetical conserved gene. (Os06t0483701-01) |  |
|  | Os06g0483900 | - | Homeodomain-like containing protein. (Os06t0483900-01) |  |
|  | Os06g0484400 | + | Conserved hypothetical protein. (Os06t0484400-01) |  |
|  | Os06g0484450 | - | Similar to Chlorophyll a-b binding protein 2 chloroplastic. (Os06t0484450-00) |  |
|  | Os06g0484500 | + | Conserved hypothetical protein. (Os06t0484500-01) |  |
|  | Os06g0484600 | + | Similar to Pherophorin-S precursor. (Os06t0484600-01) |  |
|  | Os06g0484800 | - | Reverse transcriptase domain containing protein. (Os06t0484800-00) |  |
|  | Os06g0484950 | - | MULE transposase domain containing protein. (Os06t0484950-00) |  |
|  | Os06g0485100 | - | Similar to Homeobox-like resistance. (Os06t0485100-01)%3BSimilar to Homeobox-like resistance. (Os06t0485100-02)%3BSimilar to Homeobox-like resistance. (Os06t0485100-03) |  |
|  | Os06g0486000 | - | Protein kinase core domain containing protein. (Os06t0486000-01) | OsSTA173 |
|  | Os06g0486300 | + | Similar to MLO protein homolog 1. (Os06t0486300-00) | MLO, POWDERY-MILDEW-RESISTANCE GENE O, OsMLO6 OsMLO1 OsMlo1 Mlo OsMlo-1 Mlo1, powdery-Mildew-resistance gene O6 powdery-Mildew-resistance gene O1 |
|  | Os06g0486400 | - | Serine/threonine protein kinase domain containing protein. (Os06t0486400-01) |  |
|  | Os06g0486800 | + | Similar to Formate dehydrogenase mitochondrial precursor (EC 1.2.1.2) (NAD- dependent formate dehydrogenase) (FDH). (Os06t0486800-01) | FDH, FORMATE DEHYDROGENASE, OsFDH FDH 1, Formate dehydrogenase 1 mitochondrial NAD-dependent formate dehydrogenase 1 Nad-dependent formate dehydrogenase |
|  | Os06g0486900 | + | Similar to Formate dehydrogenase mitochondrial precursor (EC 1.2.1.2) (NAD- dependent formate dehydrogenase) (FDH). (Os06t0486900-01) | FDH2 OsFDH2 |
|  | Os06g0487300 | - | Hypothetical protein. (Os06t0487300-01) |  |
|  | Os06g0487380 | + | Hypothetical gene. (Os06t0487380-01) |  |
|  | Os06g0487620 | + | Conserved hypothetical protein. (Os06t0487620-01) |  |
|  | Os06g0487660 | - | Non-protein coding transcript. (Os06t0487660-00) |  |
|  | Os06g0487700 | + | Hypothetical conserved gene. (Os06t0487700-00) |  |
|  | Os06g0487900 | - | Peptidase C48 SUMO/Sentrin/Ubl1 family protein. (Os06t0487900-01)%3BHypothetical conserved gene. (Os06t0487900-02) |  |
|  | Os06g0488050 | + | Similar to Protein kinase family protein. (Os06t0488050-00) |  |
|  | Os06g0488125 | - | Hypothetical protein. (Os06t0488125-00) |  |
|  | Os06g0488200 | + | Similar to Myosin heavy chain (Fragment). (Os06t0488200-01)%3BSimilar to XIC (Myosin-like protein XIC)%3B motor/ protein binding. (Os06t0488200-02) |  |
|  | Os06g0488600 | - | Similar to Potential phospholipid-transporting ATPase 7 (EC 3.6.3.1) (Aminophospholipid flippase 7). (Os06t0488600-01) |  |
|  | Os06g0489200 | - | Protein of unknown function DUF1604 domain containing protein. (Os06t0489200-01) |  |
|  | Os06g0489500 | + | CMP/dCMP deaminase zinc-binding domain containing protein. (Os06t0489500-01) |  |
|  | Os06g0489900 | + | Domain of unknown function DUF1618 domain containing protein. (Os06t0489900-00) |  |
|  | Os06g0490000 | - | Similar to DIMETHYLADENOSINE TRANSFERASE. (Os06t0490000-01) |  |
|  | Os06g0490200 | - | Conserved hypothetical protein. (Os06t0490200-01)%3BConserved hypothetical protein. (Os06t0490200-02)%3BConserved hypothetical protein. (Os06t0490200-03) |  |
|  | Os06g0490400 | + | Similar to Class III peroxidase 80. (Os06t0490400-00) | prx80, class III peroxidase 80 |
|  | Os06g0490700 | + | Conserved hypothetical protein. (Os06t0490700-01)%3BHypothetical conserved gene. (Os06t0490700-02) |  |
|  | Os06g0491300 | - | Hypothetical conserved gene. (Os06t0491300-01) |  |
|  | Os06g0491566 | - | Conserved hypothetical protein. (Os06t0491566-01) |  |
|  | Os06g0491800 | - | HAT dimerisation domain containing protein. (Os06t0491800-01) |  |
|  | Os06g0491901 | - | Non-protein coding transcript. (Os06t0491901-01) |  |
|  | Os06g0492000 | - | Similar to Phosphatidylinositol synthase. (Os06t0492000-01) |  |
|  | Os06g0492101 | + | Hypothetical conserved gene. (Os06t0492101-00) |  |
|  | Os06g0492300 | + | Conserved hypothetical protein. (Os06t0492300-01) |  |
|  | Os06g0492700 | + | Similar to Dynein light chain LC6 flagellar outer arm. (Os06t0492700-00) |  |
|  | Os06g0492750 | - | Hypothetical protein. (Os06t0492750-00) |  |
|  | Os06g0492800 | + | Hypothetical conserved gene. (Os06t0492800-00) | OsFbox309 Os_F0312, F-box protein 309 |
|  | Os06g0492900 | + | F-box domain cyclin-like domain containing protein. (Os06t0492900-00) | OsFbox310 Os_F0057, F-box protein 310 |
|  | Os06g0493001 | - | Hypothetical protein. (Os06t0493001-00) |  |
|  | Os06g0493100 | - | Conserved hypothetical protein. (Os06t0493100-01) | bphi008a BpHi008A, brown planthopper induced008a Brown planthopper induced 008a |
|  | Os06g0493600 | + | Phosphate (Pi) transporter Pi homeostasis (Os06t0493600-01) | PHO1%3B3, PHOSPHATE TRANSPORTER 1%3B3, OsPHO1%3B3 OsPHT1, Phosphate transporter 1 |
|  | Os06g0493700 | - | Pi homeostasis (Os06t0493700-01) | PHO1%3B3 CIS-NAT, PHOSPHATE TRANSPORTER 1%3B3 CIS-NATURAL ANTISENSE TRANSCRIPT, OsPHO1%3B3 cis-NAT |
|  | Os06g0493800 | - | Conserved hypothetical protein. (Os06t0493800-00) |  |
|  | Os06g0493801 | - | Hypothetical conserved gene. (Os06t0493801-00) |  |
|  | Os06g0493900 | + | Conserved hypothetical protein. (Os06t0493900-00) |  |
|  | Os06g0494000 | + | Paired amphipathic helix domain containing protein. (Os06t0494000-01)%3BHypothetical conserved gene. (Os06t0494000-02) |  |
|  | Os06g0494100 | - | Serine/threonine protein kinase-related domain containing protein. (Os06t0494100-01)%3BSerine/threonine protein kinase-related domain containing protein. (Os06t0494100-02) | PID2, PYRICULARIA ORYZAE RESISTANCE D2, Pi-d2 Pi-d(t)2 PiD2(t) Pid2 PI-D2 Pi-d2(t), *Pyricularia oryzae* resistance d2 *Magnaporthe grisea* resistance d2 Blast resistance d2 PI-D2 protein kinase |
|  | Os06g0494250 | + | Hypothetical protein. (Os06t0494250-00) |  |
|  | Os06g0494400 | + | Multi antimicrobial extrusion protein MatE family protein. (Os06t0494400-01) |  |
|  | Os06g0494701 | - | Conserved hypothetical protein. (Os06t0494701-01) |  |
|  | Os06g0495100 | + | Similar to cDNA clone: J100088H20 full insert sequence. (Os06t0495100-00) |  |
|  | Os06g0495500 | + | Multi antimicrobial extrusion protein MatE family protein. (Os06t0495500-01) |  |
|  | Os06g0495632 | - | Similar to F-box domain containing protein. (Os06t0495632-00) |  |
|  | Os06g0495700 | + | Beta tubulin autoregulation binding site domain containing protein. (Os06t0495700-00) |  |
|  | Os06g0495800 | + | Protein of unknown function DUF617 plant family protein. (Os06t0495800-01) |  |
|  | Os06g0496000 | + | Like-Sm ribonucleoprotein (LSM)-related domain containing protein. (Os06t0496000-01) | Sm gene Sm family protein |
|  | Os06g0496400 | + | Hypothetical conserved gene. (Os06t0496400-00) | OsbHLH127, basic helix-loop-helix protein 127 |
|  | Os06g0496601 | + | Hypothetical gene. (Os06t0496601-00) |  |
|  | Os06g0496800 | + | Similar to S-locus receptor kinase precursor. (Os06t0496800-01)%3BSimilar to S-locus receptor kinase precursor. (Os06t0496800-02) |  |
|  | Os06g0496875 | - | Non-protein coding transcript. (Os06t0496875-01) |  |
|  | Os06g0497200 | + | Hypothetical conserved gene. (Os06t0497200-00) |  |
|  | Os06g0497275 | - | Hypothetical protein. (Os06t0497275-00) |  |
|  | Os06g0497350 | + | Similar to Cytochrome P450 CYP71K14. (Os06t0497350-00) |  |
|  | Os06g0497500 | + | Conserved hypothetical protein. (Os06t0497500-00) |  |
|  | Os06g0497600 | - | Similar to H0321H01.8 protein. (Os06t0497600-01) |  |
|  | Os06g0498000 | + | Conserved hypothetical protein. (Os06t0498000-00) |  |
|  | Os06g0498150 | - | Conserved hypothetical protein. (Os06t0498150-00) | OsEnS-86, endosperm-specific gene 86 |
|  | Os06g0498400 | + | Similar to Alpha-glucan water dikinase chloroplast precursor (EC 2.7.9.4) (Starch-related R1 protein). (Os06t0498400-01)%3BSimilar to Alpha-glucan water dikinase (Fragment). (Os06t0498400-02) |  |
|  | Os06g0498450 | + | Hypothetical conserved gene. (Os06t0498450-00) |  |
|  | Os06g0498500 | + | CCAAT-binding factor domain containing protein. (Os06t0498500-01)%3BCCAAT-binding factor domain containing protein. (Os06t0498500-02) | NOC3, NUCLEOLAR COMPLEX-ASSOCIATED PROTEIN 3, Nucleolar complex-associated protein 3 |
|  | Os06g0498800 | - | Similar to MOTHER of FT and TF1 protein. (Os06t0498800-01) | OsMFT1, MFT-like gene 1 |
|  | Os06g0498900 | + | GTP-binding protein HSR1-related domain containing protein. (Os06t0498900-01) |  |
|  | Os06g0499000 | + | Conserved hypothetical protein. (Os06t0499000-01) |  |
|  | Os06g0499100 | - | Hypothetical conserved gene. (Os06t0499100-00) |  |
|  | Os06g0499200 | - | Non-protein coding transcript. (Os06t0499200-01) |  |
|  | Os06g0499301 | - | Pentatricopeptide repeat domain containing protein. (Os06t0499301-00) |  |
|  | Os06g0499500 | - | Similar to Indole-3-acetic acid-amido synthetase GH3.17 (EC 6.3.2.-) (Auxin- responsive GH3-like protein 17) (AtGH3-17). (Os06t0499500-01)%3BSimilar to GH3.17%3B indole-3-acetic acid amido synthetase. (Os06t0499500-02)%3BSimilar to Indole-3-acetic acid-amido synthetase GH3.17 (EC 6.3.2.-) (Auxin- responsive GH3-like protein 17) (AtGH3-17). (Os06t0499500-03) | GH3-7, GH3-7, OsGH3-7 |
|  | Os06g0499550 | + | Hypothetical protein. (Os06t0499550-00) |  |
|  | Os06g0499900 | - | Similar to Dihydrolipoamide acetyltransferase (E2) subunit of PDC (Fragment). (Os06t0499900-01) |  |
|  | Os06g0500100 | + | Conserved hypothetical protein. (Os06t0500100-01) |  |
| Chr7:22841126-22941126 |  |  |  |  |
|  | Os07g0568300 | + | Similar to ZF protein (Fragment). (Os07t0568300-01)%3BSimilar to ZF protein (Fragment). (Os07t0568300-02) | C3H50, ZINC FINGER CCCH DOMAIN-CONTAINING PROTEIN 50, OsC3H50 OsTZF6 OsCCCH-Zn-6, Zinc finger CCCH domain-containing protein 50 Protein ZF Tandem zinc finger protein 6 |
|  | Os07g0568400 | - | TB2/DP1 and HVA22 related protein family protein. (Os07t0568400-01) |  |
|  | Os07g0568500 | - | Peptidase aspartic active site domain containing protein. (Os07t0568500-01)%3BPeptidase aspartic active site domain containing protein. (Os07t0568500-02)%3BPeptidase aspartic active site domain containing protein. (Os07t0568500-03) |  |
|  | Os07g0568600 | - | Similar to Calcium-dependent protein kinase. (Os07t0568600-01)%3BSimilar to calcium-dependent protein kinase isoform AK1. (Os07t0568600-02) | CDPK20, CALCIUM-DEPENDENT PROTEIN KINASE 20, OsCDPK20 OsCPK20, calcium-dependent protein kinase |
|  | Os07g0568650 | + | Hypothetical protein. (Os07t0568650-00) |  |
|  | Os07g0568700 | - | Polygalacturonase inhibitor 1 precursor (Polygalacturonase-inhibiting protein) (Floral organ regulator 1). (Os07t0568700-02) | FOR1, FLORAL ORGAN REGULATOR 1, OsFOR1 Osfor1, rice floral organ regulator-1 Floral organ regulator 1 |
|  | Os07g0568800 | - | Hypothetical protein. (Os07t0568800-01) |  |
|  | Os07g0568900 | - | Conserved hypothetical protein. (Os07t0568900-01) |  |
|  | Os07g0569000 | + | Conserved hypothetical protein. (Os07t0569000-01) |  |
|  | Os07g0569100 | - | Remorin C-terminal region domain containing protein. (Os07t0569100-01) | REM4.1, REMORIN 4.1, OsREM4.1, remorin Remorin 4.1 remorin group 4 member 1 |
|  | Os07g0569166 | + | Conserved hypothetical protein. (Os07t0569166-01) |  |
|  | Os07g0569500 | + | Nicotinamide N-methyltransferase putative domain containing protein. (Os07t0569500-01) |  |
|  | Os07g0569550 | - | Similar to protein kinase. (Os07t0569550-00) |  |
|  | Os07g0569600 | - | Chaperonin-like RbcX domain containing protein. (Os07t0569600-01) |  |
|  | Os07g0569700 | + | Zinc finger C2H2-type domain containing protein. (Os07t0569700-01) | SAP16, STRESS ASSOCIATED PROTEIN GENE 16, OsSAP16, C2H2 transcription factor stress associated protein 16 |
|  | Os07g0569800 | + | Similar to protein kinase. (Os07t0569800-01) |  |
|  | Os07g0570100 | - | Similar to predicted protein. (Os07t0570100-01) |  |
| Chr7:27770508-27788464 |  |  |  |  |
|  | Os07g0659100 | - | Conserved hypothetical protein. (Os07t0659100-01) |  |
|  | Os07g0659300 | + | Conserved hypothetical protein. (Os07t0659300-01) |  |
|  | Os07g0659400 | - | Similar to rhythmically expressed protein. (Os07t0659400-00) |  |
| Chr8:496639-582447 |  |  |  |  |
|  | Os08g0109800 | + | Regulator of chromosome condensation/beta-lactamase-inhibitor protein II domain containing protein. (Os08t0109800-01) | OsCRR2, CRINKLY4-RELATED protein 2 |
|  | Os08g0109900 | - | Similar to Nucleic acid binding protein. (Os08t0109900-01) |  |
|  | Os08g0110000 | - | Alpha/beta hydrolase fold-1 domain containing protein. (Os08t0110000-01)%3BAlpha/beta hydrolase fold-1 domain containing protein. (Os08t0110000-02) |  |
|  | Os08g0110100 | + | Ribosomal protein L30 family protein. (Os08t0110100-01) |  |
|  | Os08g0110200 | + | Similar to Fertility restorer. (Os08t0110200-01) | Rf6, fertility restoration-6 RESTORATION OF FERTILITY 6 |
|  | Os08g0110300 | - | Longin domain containing protein. (Os08t0110300-01) |  |
|  | Os08g0110400 | - | Protein of unknown function DUF266 plant family protein. (Os08t0110400-01) |  |
|  | Os08g0110500 | + | U-box/ARM repeat E3 ligase Stress response Regulation of cell death Blast disease resistance (Os08t0110500-01) | OsPUB15, plant U-box-containing protein 15 U-box protein 15 Plant U-Box 15 |
|  | Os08g0110550 | - | Non-protein coding transcript. (Os08t0110550-00) |  |
|  | Os08g0110600 | - | Protein of unknown function DUF1442 domain containing protein. (Os08t0110600-00) |  |
|  | Os08g0110650 | + | Non-protein coding transcript. (Os08t0110650-00) |  |
|  | Os08g0110700 | - | DDHD domain containing protein. (Os08t0110700-01) |  |
|  | Os08g0110800 | + | K Homology type 1 subgroup domain containing protein. (Os08t0110800-01)%3BHypothetical conserved gene. (Os08t0110800-02)%3BK Homology type 1 subgroup domain containing protein. (Os08t0110800-03)%3BConserved hypothetical protein. (Os08t0110800-04)%3BHypothetical conserved gene. (Os08t0110800-05) |  |
|  | Os08g0111200 | - | Beta-glucosidase GBA2 type domain containing protein. (Os08t0111200-01)%3BSimilar to predicted protein. (Os08t0111200-02) |  |
